# Long-Term Dystrophin Replacement Therapy in Duchenne Muscular Dystrophy Causes Cardiac Inflammation

**DOI:** 10.1016/j.jacbts.2024.12.015

**Published:** 2025-03-12

**Authors:** Anne Forand, Sophie Moog, Nathalie Mougenot, Mégane Lemaitre, Caroline Sevoz-Couche, Zoheir Guesmia, Laura Virtanen, Lorenzo Giordani, Antoine Muchir, France Pietri-Rouxel

**Affiliations:** a Inovarion, Paris, France; b Centre de Recherche en Myologie-Sorbonne Université-UMRS974-Inserm-Institut de Myologie-Faculté de Médecine de la Pitié Salpêtrière, Paris, France; c Sorbonne Université-UPMC Paris 06-INSERM UMS28-Phénotypage du petit animal-Faculté de Médecine Pierre et Marie Curie, Paris, France; d Sorbonne Université-UPMC Université Paris 06-INSERM UMRS1158-Neurophysiologie Respiratoire Expérimentale et Clinique-Faculté de Médecine Pierre et Marie Curie, Paris, France; e Université Paris 12 Saclay, UVSQ-UMR 1018-CESP-Inserm-Faculté de Médecine Bicêtre, Le Kremlin-Bicêtre, France

**Keywords:** Duchenne muscular dystrophy, gene therapy, heart, inflammation, micro-dystrophin

## Abstract

Micro-dystrophin replacement gene therapy is currently under clinical trials in Duchenne muscular dystrophy (DMD) patients. However, recent adverse cardiac events has led to serious concerns about this therapeutic intervention. We studied the long-term effect of dystrophin replacement strategies in a severe model of DMD (mdx; utrophin^−/−^mice). Although micro-dystrophin remarkably improved survival and cardiac function after 1 year of treatment, we were able to reveal an increased septum thickness, which is the result of cardiac inflammation. Our data warrant consideration that micro-dystrophin replacement therapy may be associated with cardiac inflammation and opens up perspectives for understanding the consequences of using this approach in DMD patients.

## Highlights

• Micro-dystrophin gene therapy strongly increases mice survival.
• Micro-dystrophin gene therapy improves cardiac function.
• Micro-dystrophin gene therapy leads to intraventricular septum thickening due to inflammation.
• The observed phenotype is due to the transgene itself.

## Visual Abstract

**Figure:**
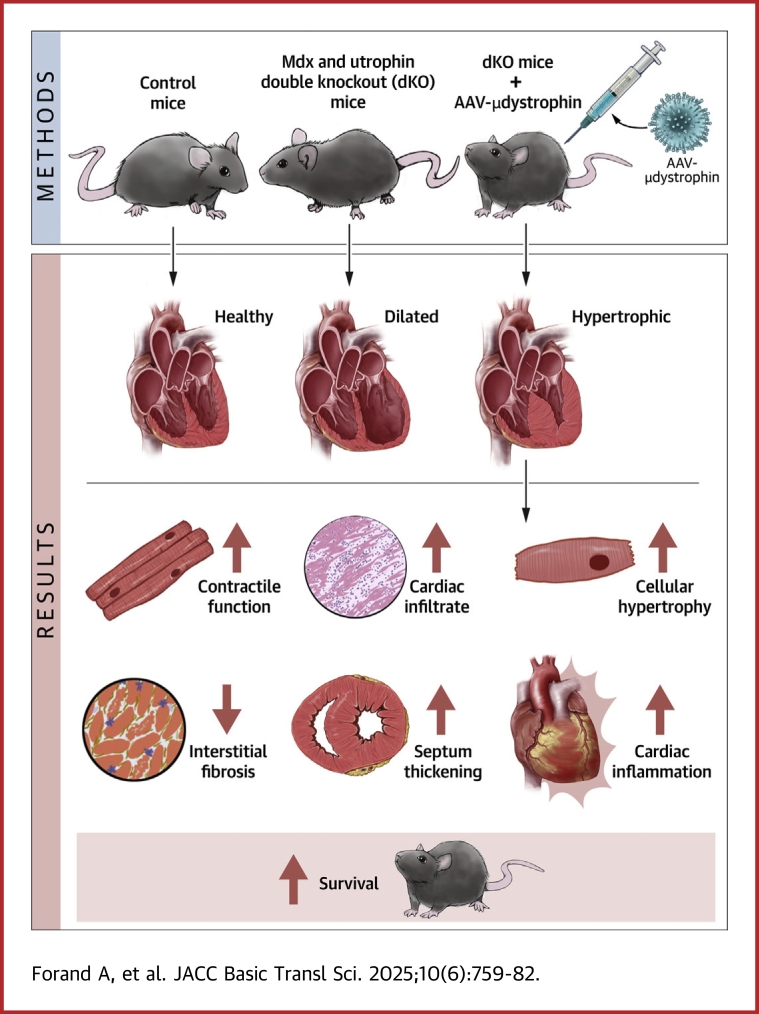

Duchenne muscular dystrophy (DMD) is a severe and progressive inherited muscular dystrophy affecting 1 in 5,000 boys in the global population.^1^^,^^2^ DMD is caused by mutations in the *DMD* gene leading to the lack of dystrophin, a structural protein located at the sarcolemma.^3^^,^^4^ Hence, the sarcolemma becomes highly susceptible to contraction-induced injury, resulting in necrosis along with cycles of degeneration and regeneration, inflammation, and appearance of fibrosis and adipose tissue within the muscle. Patients typically lose ambulation between 8 and 12 years of life and die of respiratory and/or cardiac failure in their second or third decade.^1^^,^^2^ DMD has been primarily recognized as a skeletal muscular dystrophy, and, historically, respiratory muscle insufficiencies accounted for most patient deaths. However, with improved disease management, cardiomyopathy has emerged as a leading cause of death in patients receiving assisted ventilation.^5^ Thus, development of therapeutic strategies that target skeletal and cardiac muscles is paramount for improving treatments, with the ultimate goal to cure patients.

Here we focused on the gene transfer micro-dystrophin approach, a strategy that allows targeting of skeletal and cardiac muscles and that is applicable to all DMD patients, irrespective of their mutations. Over the last 2 decades, preclinical studies showed that rescue of dystrophin expression, both by transgenic overexpression and by using exon skipping strategies, prevented skeletal muscle pathology.^6^ Some of these studies demonstrated that micro-dystrophin gene therapy displayed a higher capacity of protein expression in the heart, leading to functional benefits.^6^, ^7^, ^8^ These promising results led to clinical trials with significant improvement in disease course progression and quality of life of patients. However, more recently, serious adverse events in cardiac muscle occurred in ongoing clinical studies.^9^, ^10^, ^11^, ^12^, ^13^ With these drawbacks, it appears necessary to study the long-term impact of micro-dystrophin expression on the structure and function of cardiac muscle.

In the present study, we treated double knockout (dKO) mice, invalidated for both dystrophin and utrophin expression, with adeno-associated virus (AAV)2/9-Spc5.12-MD1 (AAV-micro-dystrophin) to elucidate the long-term effect of such treatment with a particular emphasis on cardiac tissue. After our previously published data that showed a benefit of peptide-conjugated phosphorodiamidate morpholino oligomer (PPMO) pretreatment on AAV-based gene therapy,^14^ we evaluate the effect of an additional PPMO post-treatment. Results showed that PPMO post-treatment in dKO mice, combined with PPMO pretreatment, improved skeletal muscles histology and function without an effect on cardiac tissue. In addition, our data demonstrated that AAV-micro-dystrophin treatment in dKO mice led to normal cardiac function despite a thickening of the septum associated with inflammation. Among molecular pathways identified by genome-wide microarray analysis, we pinpointed inflammatory signaling and confirmed that inflammatory cells were over-represented (B cells, macrophages, granulocytes) in the heart from wild-type (WT) and dKO mice treated with AAV-micro-dystrophin compared with untreated or AAV-backbone–treated WT mice. These data showed for the first time that micro-dystrophin gene replacement therapy in the dKO model may be associated with long-term cardiac alterations and opens up perspectives for understanding the consequences of using this approach in DMD patients.

## Methods

### Vector production

For AAV2/9-micro-dystrophin (AAV2 ITR genome with AAV9 capsid) production, a 3-plasmid (pSpc5.12MD1: micro-dystrophin transgene under the Spc5.12 promotor,^15^ pXX6, and p5E18-VD29) transfection protocol was used with PEIpro transfection reagent (Polyplus-Sartorius). AAV2/9 backbone production was obtained with transfection of pSpc5.12 without micro-dystrophin transgene, pXX6, and p5E18-VD29. Vector particles were purified from lysates obtained 48 hours after transfection, on iodixanol gradients, and titers were determined by real-time polymerase chain reaction (RT-PCR) and expressed as vector genomes per milliliter.

### Animal care

Animals were housed at the Myology Research Center (Paris, France) under 12 hours of light and 12 hours of dark conditions with ad libitum access to food and water. The experiments performed were authorized and approved by the local institutional review board (2016071210565236). All procedures conformed to the National Institutes of Health Guide for the Care and Use of Laboratory Animals.

WT C57BL/6J mice were obtained from Janvier Laboratories and injected with phosphate-buffered saline. We generated dKO and mdx mice by crossing (Utr^+/−^; Dys^−/−^) mice (JB10ScSn.Cg-Utrntm1KedDmdmdx/J; Jackson Laboratory). We injected 80 mg/kg PPMO intravenously into the temporal vein on day 2 and retro-orbitally on day 17. The dKO mice then received retro-orbital injections of 2E+12 vector genomes (corresponding to 1E+14 vector genomes per kg) of AAV2/9-micro-dystrophin (MD1)^16^ under isoflurane 4% anesthesia at the age of 3 weeks. Some of them also received a PPMO post-treatment (80 mg/kg by retro-orbital injections) at 8, 12, 16, and 20 weeks of age for the 24-week study or 70 mg/kg at 8, 16, 24, 32, and 40 weeks of age for the survival study. We selected a minimum of 8 mice (male-to-female ratio: 50/50) at random for each treatment group. Some groups were killed at 24 weeks of age (male-to-female ratio: 50/50) for molecular analysis, whereas others were retained for survival analysis until 52 weeks. The mice used for the survival analysis were killed at a humane endpoint at which their weight had decreased by more than 10% or as required by ethical rules. The WT mice received retro-orbital injections of 2E+12 vector genomes (corresponding to 1E+14 vector genomes per kg) of AAV2/9-backbone or AAV2/9-micro-dystrophin (MD1)^16^ under isoflurane 4% anesthesia at the age of 3 weeks. At the end of the procedure, mice were anesthetized with 200 μL/20 g with a mix of ketamine (100 mg/mL) and xylazine (20 mg/mL) by intraperitoneal route and killed by cervical dislocation. Cardiac and skeletal muscles were collected, snap-frozen in liquid nitrogen-cooled isopentane, and stored at −80 °C.

### Quantification of AAV genome content

Genomic DNA was extracted from mouse muscles with the Puregene Blood kit (Qiagen). AAV genome copy number and genomic DNA were determined on 100 ng of genomic DNA by absolute quantitative RT-PCR on a StepOnePlusTM system (Applied Biosystems) with *Taq*manR Universal Master Mix (Applied Biosystems). The primers (forward: CTCCATCACTAGGGGTTCCTTG and reverse: GTAGATAAGTAGCATGGC) and probe (TAGTTAATGATTAACCC) were selected for specific amplification of the vector genome sequence. As a reference sample, we generated 10-fold serial dilutions (from 10^7^ to 10^1^ copies) of a preparation of the pAAV plasmid. All genomic DNA samples were analyzed in duplicate.

### Exon skipping analysis

Total RNA was isolated from mouse muscles with NucleoSpin RNA II (Macherey-Nagel), and reverse transcription was performed on 20 ng of RNA with Superscript II and random primers (Life Technologies). Nonskipped and skipped dystrophin transcripts were detected by quantitative PCR, as previously described.^17^

### Gene expression analysis

Total RNA was isolated from mouse muscles with NucleoSpin RNA II (Macherey-Nagel), and reverse transcription was performed on 20 ng of RNA with Superscript II and random primers (Life Technologies). Quantitative RT-PCR was performed on a StepOne Plus Real-Time PCR System (Applied Biosystems) using Power SyberGreen PCR MasterMix (Applied Biosystems). All data were analyzed using the ΔΔCT method and normalized to PO (mouse acidic ribosomal phosphoprotein) mRNA expression.

### Western blot analysis

Protein extracts were obtained from pooled muscle sections treated with 125 mM sucrose, 5 mM Tris-HCl pH 6.4, 6% XT Tricine Running Buffer (Bio-Rad), 10% sodium dodecyl sulfate, 10% glycerol, and 5% β-mercaptoethanol. The samples were purified with the Pierce Compat-Able Protein Assay Preparation Reagent Set (Thermo Scientific), and total protein concentration was determined with the Pierce BCA Protein Assay Kit (Thermo Fisher Scientific). The samples were denatured by heating at 95 °C for 5 minutes, and 100 μg of protein was subjected to electrophoresis in a Criterion XT Tris-acetate precast 3% to 8% polyacrylamide gel (Bio-Rad). The membrane was probed with monoclonal primary antibodies directed against dystrophin (NCL-DYS1, 1:50 [Leica Biosystems] or Manex 1B [Glenn Moris]) and α-actinin (1:1,000; Sigma-Aldrich) and was then incubated with a goat anti-mouse secondary antibody (StarBright Blue 700-conjugated, 1:10,000; Bio-Rad) for imaging with the ChemiDoc MP Imaging System (Bio-Rad). Band intensity was quantified with Image Lab software, with the mean intensity obtained for the WT taken as 100%.

### Immunohistochemistry and histology

Frozen tissue sections (10 μm thick) were cut and stained with hematoxylin and eosin for the assessment of overall muscle morphology. Collagen fibers were detected by Sirius red staining. Sections were fixed by incubation for 10 minutes with 4% formaldehyde and dried. They were then incubated with a 0.3% solution of Sirius red in aqueous saturated picric acid for 1 hour, washed in acidified water (0.5% acetic acid), dehydrated, and mounted in VectaMount (Vector Laboratories). Fibrosis was then quantified by determining the proportion of the total cross-sectional area stained red with QuPath software.^18^ First, we imported the histologic images into the software and then trained a QuPath artificial neural network model to define 2 regions of interest with red-stained collagen and fibers. Finally, we quantified the collagen content by measuring the positive staining within the regions of interest and exported the data for statistical analysis and interpretation.

### Immunofluorescence

Frozen tissue sections (10 μm thick) were cut and incubated with permeabilization solution (phosphate-buffered saline–0.5% Triton) for 10 minutes and then blocking solution (phosphate-buffered saline–5% bovine serum albumin–0.1% Triton) for 30 minutes. Primary antibodies directed against dystrophin (NCL-DYS1, 1:50 [Leica Biosystems] or Manex 1B [Glenn Moris]), α-actinin (Sigma A2172), and/or laminin (Sigma L9393) were diluted in blocking solution and incubated overnight at 4 °C and were then incubated with Alexa coupled secondary antibody for 1 hour at room temperature. Nuclei were counterstained with DAPI.

### Muscle function assessments

Specific maximal force and force drop (a measure of susceptibility to contraction-induced injury) were evaluated by measuring the contraction of the tibialis anterior (TA) muscle in response to nerve in situ, as previously described.^19^ The investigators were blinded to treatment allocation and outcome assessment. Mice were anesthetized with 70 mg/kg body weight of pentobarbital. Body temperature was maintained at 37 °C with radiant heat. The knee and foot were fixed with pins and clamps, and the distal tendon of the muscle was attached to a lever arm in a servomotor system (305B, Dual-Mode Lever; Aurora Scientific) with a silk ligature. The sciatic nerve was proximally crushed and distally stimulated by a bipolar silver electrode, with supramaximal square wave pulses of 0.1-ms duration (10 V). We measured the absolute maximal force generated during isometric tetanic contractions in response to electrical stimulation (150 Hz, 500 ms). Absolute maximal force was determined at L0 (the length at which maximal tension was obtained during the tetanus). Absolute maximal force was normalized against muscle weight as an estimate of specific maximal force (absolute maximal force/muscle weight).

Susceptibility to contraction-induced injury was estimated by determining the force drop resulting from lengthening contraction-induced injury. The sciatic nerve was stimulated for 700 ms (frequency of 125 Hz). A maximal isometric contraction of the TA muscle was initiated during the first 500 ms. Muscle lengthening (10% L0), at a velocity of 5.5 mm/s (0.85 fiber lengths/s), was then imposed during the last 200 ms. Nine lengthening contractions of the muscle were performed, each separated by a 45-second rest period. All contractions were performed at an initial length L0. The data were recorded with the Powerlab 8/36 data acquisition system (AD Instruments). Maximal force was measured for each contraction and expressed as a percentage of the initial maximal force.

### Echocardiography

Mice were lightly anesthetized with 0.2% to 0.5% isoflurane in O_2_ and placed on a heating pad (37 °C). Echocardiography was performed with a probe emitting ultrasound at a frequency of 9 to 14 MHz (Vivid7 PRO apparatus; GE Medical System Co) applied to the chest wall. Cardiac ventricular dimensions and fractional shortening were measured in the 2-dimensional mode and M-mode. Examinations were performed by an echocardiographer blinded to genotype and treatment.

### Electrocardiography

Electrocardiograms were recorded for the mice with the noninvasive electrocardiography (ECG) TUNNEL (Emka Technologies) with minimal filtering. Waveforms were recorded with Iox software, and intervals were measured manually with ECG auto software (Emka). The investigators were blinded to treatment allocation and outcome assessment.

### Measurement of ventilation

Ventilation was measured in a blinded manner by whole-body flow-through plethysmography. The measurement and reference chambers were connected to a pneumotachograph (Fleisch 0000) connected to a volume transducer (Gould Z46170). Respiratory signals were relayed to a 1401 interface (1401 Plus; CED) and processed with Spike 2 Software (6.14; CED). Both respiratory frequency and tidal volume were derived from the ventilatory flow signal. Minute ventilation was calculated as follows: V_E_ = V_T_ × *f*R, where VE is the ventilation, *f*R is the respiratory frequency and V_T_ is the tidal volume.

### Creatine kinase assay

Creatine kinase levels were measured in mice sera using a creatine kinase activity assay kit (Sigma) according to manufacturer’s instructions.

### IMC

#### Staining

Three tissue sections for each condition were used for the imaging mass cytometry (IMC) in 2 separate experiments. Tissue sections were fixed with 4% paraformaldehyde (15735-90; Electron Microscopy Science) at room temperature for 10 minutes and permeabilized with prechilled MetOH at –20 °C for 6 minutes followed by blocking in 3% bovine serum albumin (HALALB07-62; Eurobio) for 45 minutes. The first set of metal conjugated antibodies were incubated overnight at +4 °C in 0.5% bovine serum albumin, and the second set was incubated the following day at room temperature for 2 hours in 0.5% bovine serum albumin (Supplemental Table 1). Slides were washed 3 times with 0.2% Triton X-100 in phosphate-buffered saline before incubation with Cell-ID intercalator-Ir (1:800, 201192A; Standard Biotools) for 30 minutes at room temperature to visualize DNA. After washing twice in doubly distilled H_2_O, slides were left to dry before imaging.

#### Imaging

Images were acquired using a Helios time-of-flight mass cytometer coupled to a Hyperion Imaging System (Standard Biotools). Before laser ablation, optical images of slides (called panorama) were acquired using the Hyperion software, and the areas to ablate were selected as described below. Laser ablation was performed at a resolution of approximately 1 μm and a frequency of 200 Hz. Upon staining, approximately 1 mm × 1 mm region was analyzed from the septum area of each heart section by IMC using the Hyperion system (Standard Biotools).

#### Spillover compensation

Although spectral overlap between pure metal isotopes is very limited, channel crosstalk caused by isotopic impurity, oxide formation, and imprecision in ion detection still exists. Spillover compensation was performed as previously described.^20^ Briefly, an agarose-coated microscopy slide was spotted with the same antibodies–metals couples used in the panel (Supplemental Table 1) (1 metal isotope per spot). With this strategy, each metal is measured individually while having all channels open for detection, allowing measure crosstalk from 1 channel to the other and for spillover quantification across all measured channels. R package CATALYST^20^ was used to convert the resulting .txt files to the spillover matrix. The spillover matrix was combined with the single cell data in the SpatialExperiment object to correct expression values.

#### Image preprocessing

Cell segmentation was performed using Visiopharm software. Visiopharm reads mcd files natively and was subsequently used to define cell masks using a deep learning method. The pixels in each image were defined as belonging to the nucleus, border (cytoplasm/membrane), or background compartment using the pixel classification module. Training of the U-Net classifier was performed on 3 to 4 region of interest substacks generated from the original images and containing only informative markers (iridium). This allowed for the generation of maps that integrate for each pixel the probability of belonging to each of 3 compartments. Based on the trained classifier, probability maps were generated for the whole dataset. To define cell borders, nuclei were first identified as primary objects based on Visiopharm probability maps and applied a 3-pixel expansion, and the masks were exported as tiff files in batch mode.

The MCD viewer (Standard Biotools) was used to convert raw image data into 1-tiff formats. Images were preprocessed with medium filtering using ImageJ Fiji^21^ for the hot pixel removal.^22^ Preprocessed images and nuclear masks were then imported into HistoCAT where expression counts of individual cells of each channel were acquired and exported as an Excel sheet (Microsoft).

#### Image analysis

The R package imcRtools was used for the data analysis as previously described.^23^ Briefly, a SpatialExperiment object was created from the single-cell data generated by HistoCAT. After loading the single-cell data, some additional processing steps were required. The distribution of expression counts across cells were skewed toward the right side; therefore, the expression counts were transformed using an inverse hyperbolic sine function (asinh(1/counts)). The batch effect between samples and experiments were corrected using the R package harmony.^24^ Cells were clustered using the R package bluster and shared the nearest-neighbor graph approach. Cell clusters were manually annotated based on marker expressions. Neutrophils were selected using Ly6G expression, macrophages based on CD11b expression, and the remaining immune cells were selected based on expression levels of MHCII, CD86, F480, CD54, and CD24. Because of the close proximity of endothelial cells and pericytes, they were clustered together using markers CD31 and CD146. The antibody panel was designed to analyze the presence of the immune cells in the heart septum and was not meant for detailed analysis of different classes of immune cells or other cell types (eg, fibroblasts, cardiomyocytes, and smooth muscle cells).

### RNA sequencing and analysis

RNA was isolated from 52-week-old WT and dKO mice treated with AAV-micro-dystrophin using the RNA Easy Mini Kit (Qiagen) according to the manufacturer’s instructions. To reduce genomic DNA contamination, a 15-minute on-column DNase I treatment at room temperature was performed. RNA samples from 5 mice from each group were submitted to Illumina DRAGEN bio-IT Platform (v3.10.4) for RNA sequencing and analysis. Purified RNAs were quantified with NanoDrop, and the quality of the RNA was assessed using the High Sensitivity RNA ScreenTape on the Agilent 2100 Bioanalyzer (Agilent Technologies). All RNA had an RNA integrity number > 8.0 and was of high quality. Quality of raw data was evaluated with FastQC. Poor-quality sequences and adapters were trimmed or removed with Dragen, with default parameters, to retain only good-quality paired reads. Illumina DRAGEN bio-IT Platform (v3.10.4) was used for mapping on mm10 reference genome and quantification with gencode vM25 annotation gtf file. Library orientation, library composition, and coverage along transcripts were checked with Picard tools. Analyses were conducted with R software.^25^ Data were normalized with DESeq2 (v1.26.0) bioconductor packages before differential analysis with DESeq2 workflow. Multiple hypothesis–adjusted *P* values were calculated with the Benjamini-Hochberg procedure to control the false discovery rate. Finally, enrichment analysis was conducted with the clusterProfiler R package (v3.14.3) with Gene Set Enrichment Analysis on the Hallmark gene set database.

### Statistical analysis

Results are presented in the figures using actual data points and the mean ± SEM. Comparisons among >2 groups were assessed by 1-way analysis of variance with Dunnett's or Tukey's post-hoc test if normally distributed or the Kruskal-Wallis test otherwise. Repeated-measures analysis of variance was used to compare body weight groups over time. Differences between 2 groups were assessed using unpaired Student *t* tests or the Mann-Whitney U test as appropriate based on distribution. Welch's correction was applied if the assumption of homogeneous variances was not confirmed. The association between continuous variables was assessed using Pearson's correlation coefficient (r). Survival among groups was evaluated with Kaplan-Meier curves (log-rank test). A *P* < 0.05 was considered statistically significant. Normal distribution was assessed using the Shapiro-Wilk test. Statistical analyses were performed using GraphPad Prism software 10.0.

## Results

### AAV-micro-dystrophin approach is efficient for extending survival of the severe DMD mouse model dystrophin^−/−^; utrophin^−/−^

**Figure 1:**
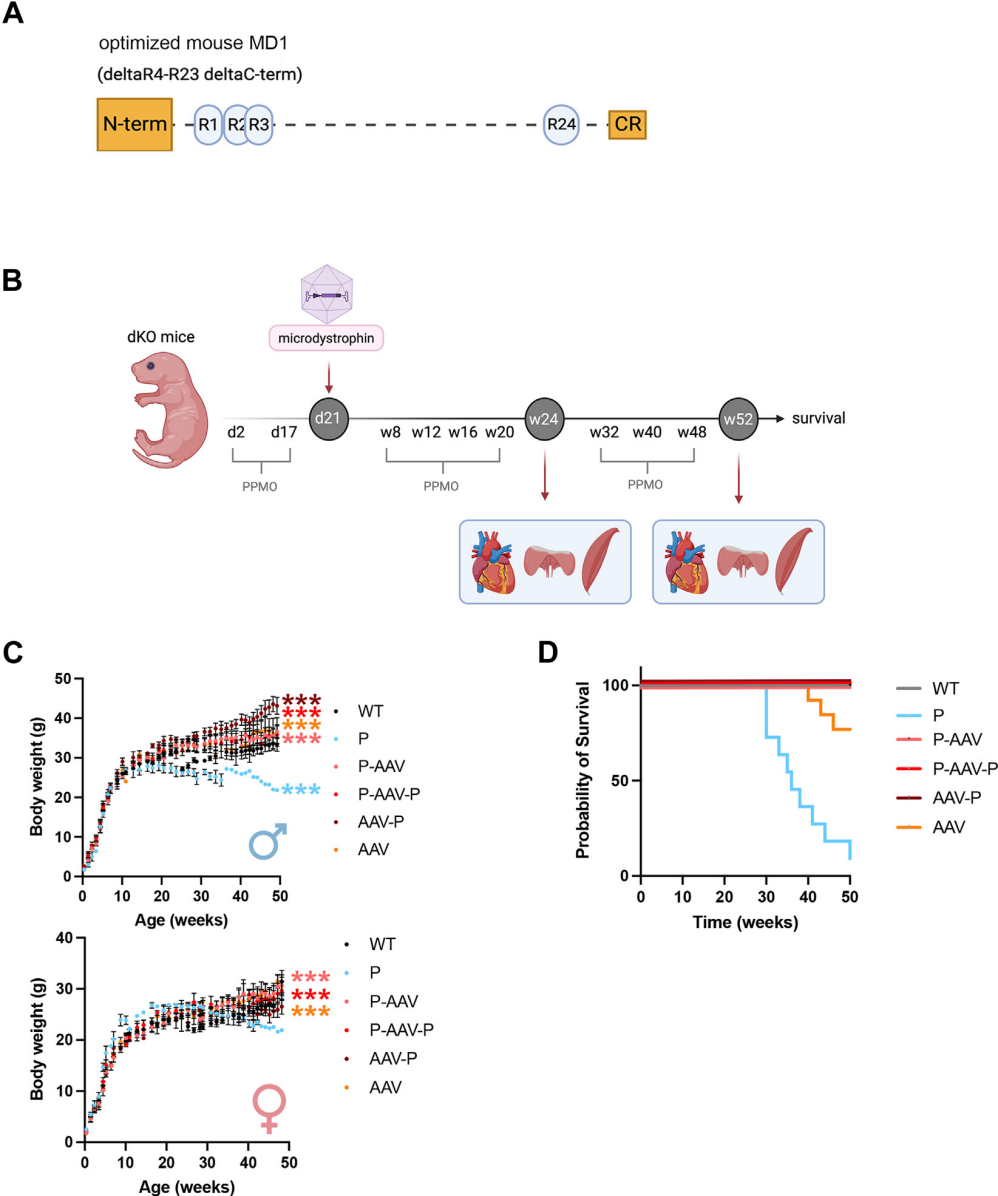
AAV-Micro-Dystrophin Treatment Rescue Body Weight Gain and Increase Survival of dKO Mice (A) Schematic representation of the optimized mouse micro-dystrophin MD1 under control of the Sp5.12 promotor. (B) Schematic representation of the double knockout (dKO) systemic treatments with peptide-conjugated phosphorodiamidate morpholino oligomer (PPMO) and AAV-micro-dystrophin. We injected 80 mg/kg PPMO intravenously into the temporal vein on day 2 and retro-orbitally on day 17. The dKO mice then received retro-orbital injections of 2E+12 vector genomes (corresponding to 1E+14 vector genomes per kg) of AAV2/9-μdystrophin (MD1) under general anesthesia, at the age of 3 weeks. Some of them also received PPMO post-treatment (80 mg/kg by retro-orbital injections) at 8, 12, 16, and 20 weeks of age for the 24-week study or 70 mg/kg at 8, 16-, 24-, 32- and 40-weeks old for the survival study. (C) Over time body weight comparisons between wild-type (WT) mice and dKO mice treated with PPMO alone (P), PPMO+AAV-micro-dystrophin (P-AAV), PPMO+AAV-micro-dystrophin+PPMO (P-AAV-P), AAV-micro-dystrophin+PPMO (AAV-P), and AAV-micro-dystrophin alone (AAV), along the course of the disease. Data are means ± SEM of at least 4 mice per group. Statistical significance was determined by repeated 1-way analysis of variance (with Dunnett’s multiple comparison test with WT) (∗∗∗*P* < 0.001, relative to WT mice). (D) Survival rate of WT mice and dKO mice treated with PPMO alone (P), PPMO+AAV-micro-dystrophin (P-AAV), PPMO+AAV-micro-dystrophin+PPMO (P-AAV-P), AAV-micro-dystrophin+PPMO (AAV-P), and AAV-micro-dystrophin alone (AAV). Groups are composed of at least 8 animals. Survival curves of WT mice and dKO mice treated with P-AAV, P-AAV-P, and AAV-P are superimposed and reached 100%. The other survival curves (P and AAV) are significantly different, log rank test. (Parts of the figure were drawn by using BioRender.com.) AAV = adeno-associated virus.

The dKO mouse model represents a translational bridge toward humans because it mimics more closely the human disease than other existing murine models of dystrophin deficiency.^26^ Indeed, dKO mice share the same pathogenesis as human disease^27^ and are a more severe phenotype than the *mdx* mouse and therefore are considered a more relevant model for identification of therapeutic strategies. We demonstrated a striking benefit of a combined therapy with AAV-U7 treatment and PPMO on striated muscles in dKO mice with significant extent of survival.^14^ This proof of principle performed; we evaluated benefits of sequential treatments using micro-dystrophin administered by systemic route in 3-week-old dKO mice (Figure 1A). For this purpose, we injected dKO mice during the juvenile period and compared survival and cardiac outcomes with untreated WT mice, given that untreated dKO mice died around 9 weeks of age (Figure 1B). The AAV-micro-dystrophin treatment and the different combined therapies restored normal weight gain in both dKO male and female mice compared with untreated WT mice at 52 weeks of age (Figure 1C). dKO mice receiving only 2 PPMO injections (2 and 17 days) without AAV-micro-dystrophin always showed the least improvement. Although dKO mice died prematurely (median survival: 7 weeks),^14^ mice that received PPMO pretreatment alone died later (median survival: 36 weeks), and survival was remarkably improved with the AAV-micro-dystrophin treatment and combinations (median survival > 52 weeks) (Figure 1D).

### AAV-micro-dystrophin approach is efficient for expressing micro-dystrophin in skeletal muscles of dKO mice and partially restores their functions

We then explored the effect of AAV-micro-dystrophin expression on diaphragm and TA skeletal muscles. AAV genomes were found only in groups of dKO mice receiving AAV-micro-dystrophin (Supplemental Figure 1A, Supplemental Figure 2A). Increased micro-dystrophin levels were observed in all the different animal groups at 24 weeks and 52 weeks in skeletal muscles from AAV-micro-dystrophin treated mice compared with age-related WT mice as well as 9-week-old dKO mice, both at RNA (Supplemental Figure 1B, Supplemental Figure 2B) and protein expression levels (Supplemental Figures 1C and 1D, Supplemental Figures 2C and 2D). We then quantified PPMO efficiency to skip *Dmd* gene exon 23 and restore the mRNA open reading frame. We found skipped *Dmd* transcript in dKO mice receiving the PPMO post-treatment only (Supplemental Figure 1E, Supplemental Figure 2E). Accordingly, dystrophin protein rescue was detectable only in the skeletal muscles of the post-treated mice. Data also suggest that PPMO efficiency to restore dystrophin expression is lost over time without readministration.

We further evaluated the benefit of AAV-micro-dystrophin treatment and the different combined therapies on skeletal muscle structure and function. We observed increased levels of creatine kinase in the PPMO, PPMO-AAV, and AAV groups (Supplemental Figure 3A), which is a feature of skeletal muscle injury. TA muscle weight was increased in the PPMO group, whereas it was decreased in all other groups compared with WT (Supplemental Figure 3B). The structure of TA muscles (Supplemental Figure 3C) showed central nuclei (Supplemental Figure 3D), high levels of infiltration (Supplemental Figure 3E), and fibrosis (Supplemental Figure 3F) in the PPMO group at 24 weeks. Central nuclei (shown as a percentage in Supplemental Figure 3D) were also found in AAV-PPMO and AAV groups and to a lesser extent in the PPMO-AAV group. At 52 weeks, TA muscle from dKO AAV-treated groups displayed a percentage of central nucleated fibers similar to WT mice, also for the AAV-PPMO group (Supplemental Figure 3D). In addition, we observed a significant increase of interstitial nuclei amount in TA muscle from AAV-PPMO and AAV groups at 52 weeks. Altogether, these observations suggest that PPMO pretreatment was beneficial to preserve TA muscle integrity and to prevent inflammation. In addition, a decreased specific maximal force and an increased fragility were observed for PPMO, PPMO-AAV, and AAV groups at 24 weeks (Supplemental Figures 3G and 3H), suggesting that PPMO post-treatment is beneficial for TA muscle structure and function.

Considering the structure of diaphragm muscle, histologic study showed dramatic alterations at 24 weeks of age in the PPMO and the AAV-PPMO groups with high rates of central nuclei and high level of tissue infiltration by leukocytes (CD45^+^ cells) (Supplemental Figure 4A). At 52 weeks of age, we could observe the onset of lipid droplets, correlated with a trend toward increased *perilipin* mRNA expression in all treated groups (Supplemental Figure 4B). Infiltration in the AAV-PPMO group was identified as sign of inflammation given that leukocyte (CD45) markers were increased (Supplemental Figure 4B). This correlates with an increased fibrosis in the diaphragm (Supplemental Figures 4C and 4D). Ratios of diaphragm weight to body weight were normalized for all treated mice compared with WT mice (Supplemental Figure 5A). The respiratory rate was increased in the PPMO group at 24 weeks, but this was compensated by a slight decreased tidal volume because the ventilation was not significantly altered in comparison with WT mice (Supplemental Figure 5B). The same observation was done at 52 weeks in the AAV-PPMO group. Taken together, these results suggest that PPMO treatment is beneficial for the diaphragm but only when used as a combination pretreatment.

### AAV-micro-dystrophin is efficient for expressing micro-dystrophin in the heart and improving cardiac muscle function

The cogency of our approach was assessed by reporting the cardiac viral genome content in injected animals. Viral genome was found only in groups of dKO mice receiving AAV-micro-dystrophin (Supplemental Figure 6A). The efficacy of the treatment was then evaluated by quantifying dystrophin levels in all the different animal groups at 24 weeks and 52 weeks. Increased micro-dystrophin levels were observed in the heart from AAV-micro-dystrophin–treated mice compared with age-related WT mice as well as 9-week-old dKO mice, both at RNA (Supplemental Figure 6B) and protein expression level (Supplemental Figures 6C and 6D), as well as protein localization at the sarcolemma (Supplemental Figures 6E and 6F). At 52 weeks of age, micro-dystrophin was expressed in almost all ventricular cardiomyocytes, with heterogeneous levels of expression (Supplemental Figure 6F). We also investigated PPMO efficiency in heart. Skipped exon 23 dystrophin mRNA expression was found only in groups of dKO mice receiving the PPMO post- treatment (Supplemental Figure 7A). As expected, no skipped exon 23 dystrophin mRNA expression was observed in the pretreated groups of mice at 24 and 52 weeks because of the clearance of the PPMO. Accordingly, dystrophin protein (skipped for exon 23) was weakly detectable only in the hearts of the post-treated groups (Supplemental Figures 7B and 7C).

**Figure 2:**
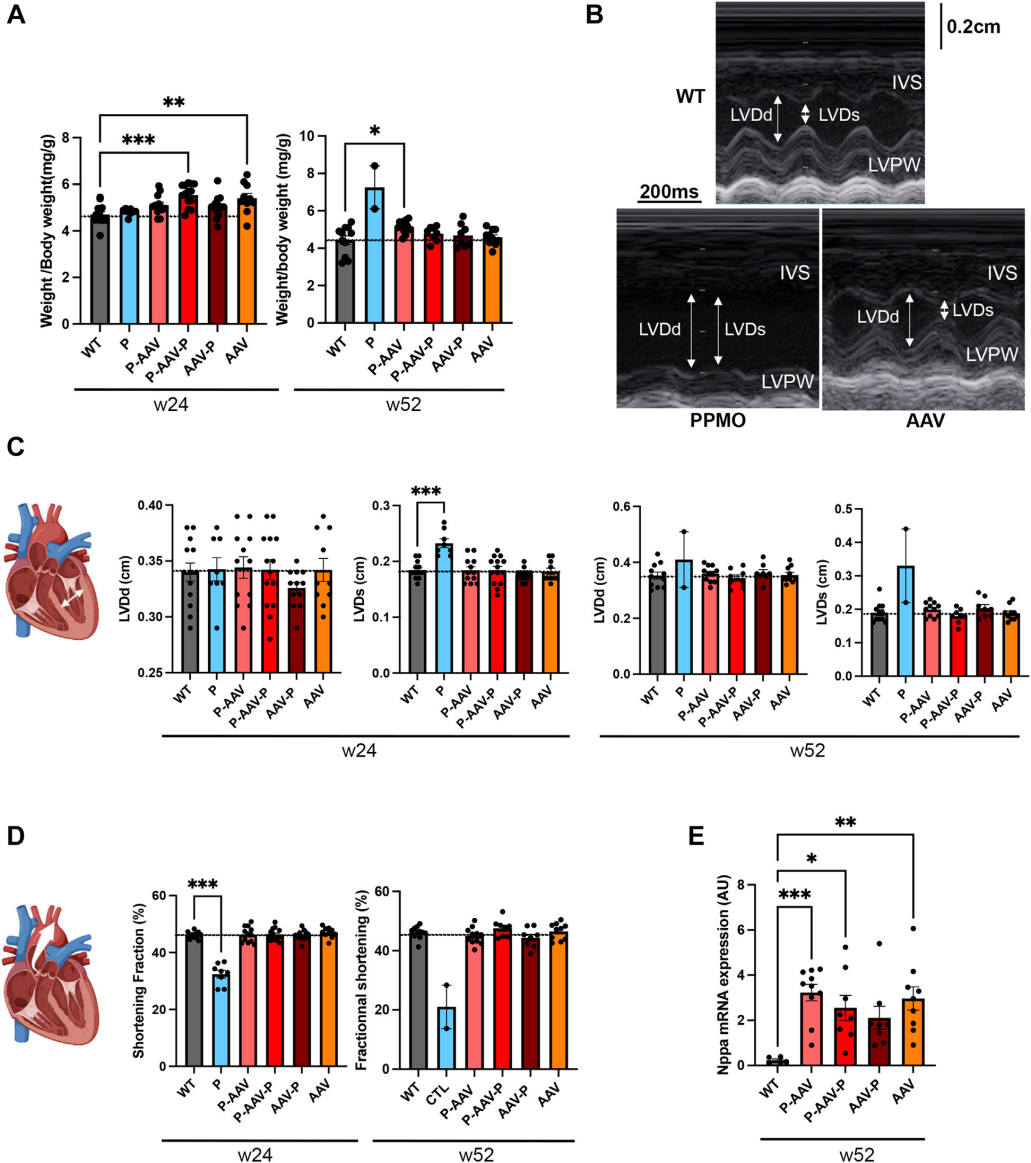
AAV-Micro-Dystrophin Treatment Improves Cardiac Function in dKO Mice (A) Heart weight-to-body weight ratio calculated from wild-type (WT) mice and double knockout (dKO) mice treated with peptide-conjugated phosphorodiamidate morpholino oligomer (PPMO) alone (P), PPMO+AAV-micro-dystrophin (P-AAV), PPMO+AAV-micro-dystrophin+PPMO (P-AAV-P), AAV-micro-dystrophin+PPMO (AAV-P), and AAV-micro-dystrophin alone (AAV) at weeks 24 and 52. (B) Representative M-mode transthoracic echocardiographic tracings from 52-week-old WT mice and dKO mice treated with PPMO or AAV alone. (C) Bar graphs showing the left ventricular end-diastolic diameters (LVDd) and end-systolic diameters (LVDs) in WT mice and dKO mice treated with PPMO alone (P), PPMO+AAV-micro-dystrophin (P-AAV), PPMO+AAV-micro-dystrophin+PPMO (P-AAV-P), AAV-micro-dystrophin+PPMO (AAV-P), and AAV-micro-dystrophin alone (AAV) at weeks 24 and 52. (D) Bar graphs showing the fractional shortening in WT mice and dKO mice treated with PPMO alone (P), PPMO+AAV-micro-dystrophin (P-AAV), PPMO+AAV-micro-dystrophin+PPMO (P-AAV-P), AAV-micro-dystrophin+PPMO (AAV-P), and AAV-micro-dystrophin alone (AAV) at weeks 24 and 52. (A and B) Data are means ± SEM of at least 8 mice per group except for the PPMO group at 52 weeks, which is means ± SEM of 2 mice 48 weeks old. Significance was determined by 1-way analysis of variance (with Dunnett’s multiple comparison test to WT). (E) Bar graphs showing quantitative real-time polymerase chain reaction analysis of *Nppa* expression in the hearts of 52-week-old WT mice and dKO mice treated with PPMO alone (P), PPMO+AAV-micro-dystrophin (P-AAV), PPMO+AAV-micro-dystrophin+PPMO (P-AAV-P), AAV-micro-dystrophin+PPMO (AAV-P), and AAV-micro-dystrophin alone (AAV). Of note: the PPMO group includes mice aged 38 to 50 weeks. Data are means ± SEM for at least 5 mice per group, and significance was determined by 1-way analysis of variance with Dunnett’s multiple comparison test to WT. ∗*P* < 0.05, ∗∗*P* < 0.01, ∗∗∗*P* < 0.001. Abbreviation as in Figure 1.

We further evaluated the benefit of AAV-micro-dystrophin treatment on cardiac function. The ratio of heart weight to body weight tended to increase at 24 weeks in all AAV-treated mice compared with WT mice (Figure 2A). Left ventricular end-systolic and diastolic diameters were restored in dKO mice to a level similar to WT mice in all groups receiving AAV-micro-dystrophin (Figures 2B and 2C, Supplemental Table 2). Cardiac function was also improved in AAV-micro-dystrophin–treated dKO mice, as gauged by ejection fraction and fractional shortening values comparable with WT mice (Figure 2D, Supplemental Table 2). PPMO treatment alone had no beneficial effect on cardiac dilation and contractile function. Given that PPMO showed a weak efficacy in the heart,^28^^,^^29^ dKO mice treated with only PPMO recapitulated the progression toward heart failure characteristic of DMD cardiomyopathy.^30^ *Nppa*, which encodes atrial natriuretic precursor, expression level represents a useful diagnostic marker for heart failure. The expression of *Nppa* gene was increased (10%) in hearts of mice receiving AAV-micro-dystrophin compared with WT mice (Figure 2E). Given that *Nppa* mRNA was reported to be increased by 50-fold in 9-week-old dKO mice compared with age-related WT mice,^14^ we could extrapolate that treatments significantly halted the *Nppa* expression level in the mouse model of DMD.

**Figure 3:**
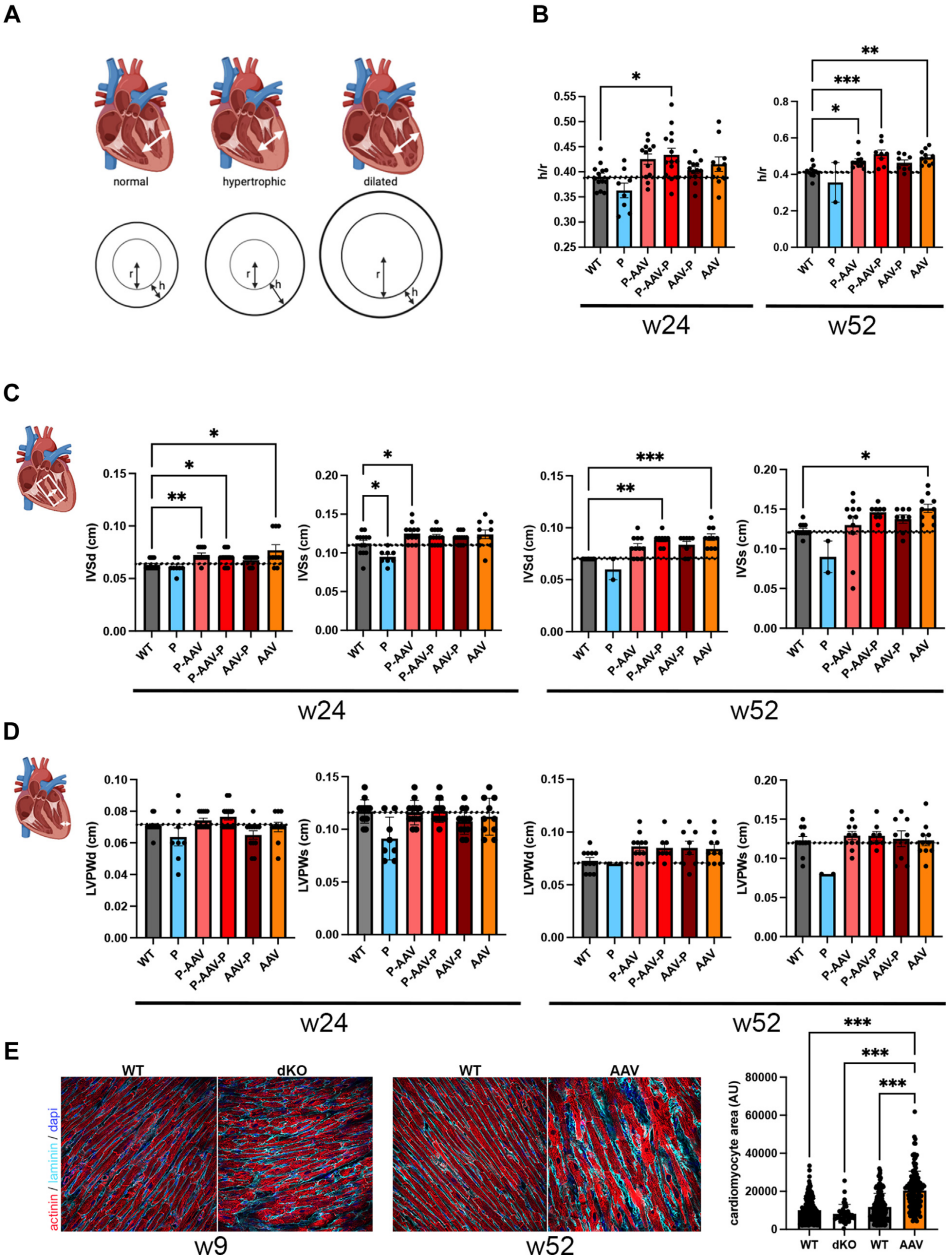
dKO Mice Treated With AAV-Micro-Dystrophin Have Compensatory Cardiac Hypertrophy (A) Schematic significance of h/r ratio modulation. (B) Bar graphs showing the h/r ratio in 24-week-old and 52-week-old wild type (WT) and double knockout (dKO) mice treated with peptide-conjugated phosphorodiamidate morpholino oligomer (PPMO) alone (P), PPMO+AAV-micro-dystrophin (P-AAV), PPMO+AAV-micro-dystrophin+PPMO (P-AAV-P), AAV-micro-dystrophin+PPMO (AAV-P), and AAV-micro-dystrophin alone (AAV). (C) Bar graphs showing the intraventricular end-diastolic thickness (IVSd) and end-systolic thickness (IVSs) in WT mice and dKO mice treated with PPMO alone (P), PPMO+AAV-micro-dystrophin (P-AAV), PPMO+AAV-micro-dystrophin+PPMO (P-AAV-P), AAV-micro-dystrophin+PPMO (AAV-P), and AAV-micro-dystrophin alone (AAV), at weeks 24 and 52. (D) Bar graphs showing the left ventricular posterior wall thickness in diastole (LVPWd) and systole (LVPWs) in WT mice and dKO mice treated with PPMO alone (P), PPMO+AAV-micro-dystrophin (P-AAV), PPMO+AAV-micro-dystrophin+PPMO (P-AAV-P), AAV-micro-dystrophin+PPMO (AAV-P), and AAV-micro-dystrophin alone (AAV), at weeks 24 and 52. Data are means ± SEM of at least 8 mice per group except for the PPMO group at 52 weeks, which is means ± SEM of 2 mice 48 weeks old. Significance was determined by 1-way analysis of variance when data were normally distributed or Kruskal-Wallis when they were not normally distributed (Dunnett’s comparison with WT). (∗*P* < 0.05, ∗∗*P* < 0.01, ∗∗∗*P* < 0.001). (E) Micrographs showing Laminin (blue) and α-actinin (red) staining in hearts from 9-week-old WT and untreated dKO mice (left panel) and 52-week-old WT mice and dKO mice treated with AAV-micro-dystrophin alone (AAV) (right panel). Scale bar: 10 μm. Bar graphs showed the cardiomyocyte area (means ± SEM) quantified with QuPath software. Significance was determined with analysis of variance with multiple comparisons. Abbreviation as in Figure 1.

We next calculated the ratio between thickness (h) of the ventricular wall and the ventricular lumen radius (r) (h/r ratio) by echocardiography, which is an important information for heart disease. Indeed, left ventricular wall stress is directly proportional to lumen pressure and radius and is inversely proportional to wall thickness (Figure 3A). We reported an increased h/r ratio in dKO mice treated with AAV-micro-dystrophin compared with untreated WT mice (Figure 3B). An increased h/r ratio associated with a normal ejection fraction could reflect a concentric remodeling.^31^ This was associated with increased diastolic and systolic intraventricular septum in dKO mice treated with AAV-micro-dystrophin compared with WT mice (Figure 3C). An interventricular septum thickness has been reported to serve as a prediction for cardiovascular diseases.^32^ A slight increase in the left ventricular posterior wall in diastole was reported in treated dKO mice at 24 weeks, compared with WT mice (Figure 3D). The increase in intraventricular septum thickness was associated with microscopic cardiomyocyte hypertrophy (Figure 3E).

**Figure 4:**
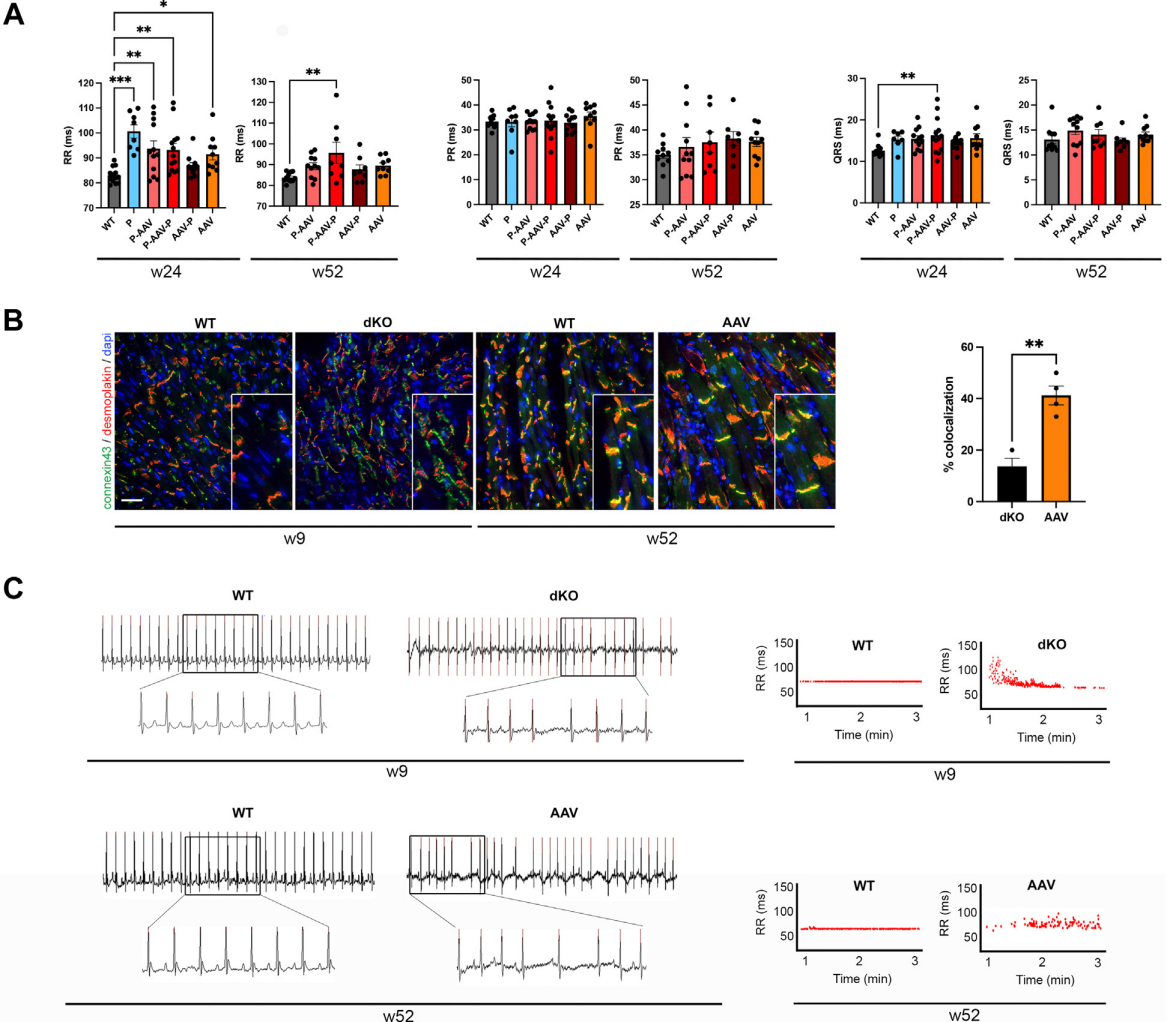
dKO Mice Treated With AAV-Micro-Dystrophin Show Minor Arrhythmic Events Despite Normal Electrocardiographic and Correct Connexin-43 Localization (A) Bar graphs showing the electrocardiographic data with RR duration (left panel), PR duration (medium panel), and QRS duration (right panel) in wild-type (WT) mice and double knockout (dKO) mice treated with peptide-conjugated phosphorodiamidate morpholino oligomer (PPMO) alone (P), PPMO+AAV-micro-dystrophin (P-AAV), PPMO+AAV-micro-dystrophin+PPMO (P-AAV-P), AAV-micro-dystrophin+PPMO (AAV-P), and AAV-micro-dystrophin alone (AAV) at weeks 24 and 52. Data are means ± SEM of at least 8 mice per group. Significance was determined by 1-way analysis of variance (Dunnett’s multiple comparison with WT). (B) Micrographs showing connexin-43 (green) and desmoplakin (red) staining in hearts from 9-week-old WT and untreated dKO mice (left panel) and 52-week-old WT mice and dKO mice treated with AAV-micro-dystrophin alone (AAV) (right panel). Scale bar: 10 μm. Bar graphs showed connexin-43 and desmoplakin colocalization percentage (means ± SEM) quantified with QuPath software. Significance was determined with the Mann-Whitney test. (C) Graphs showing RR duration over time in 9-week-old WT and untreated dKO mice (left panel) and 52-week-old WT mice and dKO mice treated with AAV-micro-dystrophin alone (AAV) (right panel). ∗*P* < 0.05, ∗∗*P* < 0.01, ∗∗∗*P* < 0.001. Abbreviation as in Figure 1.

Given that dKO mice display abnormal ECG results and that DMD patients develop arrhythmias and cardiac conduction defects, we next monitored the cardiac electrical activity of the mice by ECG. Data revealed that dKO mice treated with AAV-micro-dystrophin have minor cardiac conduction defects (longer RR and QRS interval for some treated animals) at 52 weeks compared with age-matched WT mice (Figure 4A, Supplemental Table 3). Changes in the ECG interval have been correlated with remodeling of connexin-43 in the hearts of mdx and dKO mice as well as in the hearts of DMD patients.^33^ We hypothesized that a restoration of connexin-43 at the cell-to-cell junction was associated with a proper cardiac conduction in dKO mice treated with AAV-micro-dystrophin. By fluorescent microscopy analysis, we showed that connexin-43 was not properly localized at intercalated disks in cardiomyocytes from dKO mice at 9 weeks of age (Figure 4B). After 52 weeks of AAV-micro-dystrophin treatment, connexin-43 was well located at intercalated disks (Figure 4B). Given that DMD patients and dKO mice are at risk for ventricular arrhythmia leading to sudden cardiac death, we therefore searched for such defects in dKO mice treated with AAV micro-dystrophin. We were able to identify arrhythmic events, defined as the variation of the RR interval, per minute, whereas untreated WT animals showed no alteration. We did not detect ventricular fibrillation or second- and third-degree heart blocks in injected dKO mice (Figure 4C).

### AAV-micro-dystrophin is associated with inflammation in cardiac muscle

**Figure 5:**
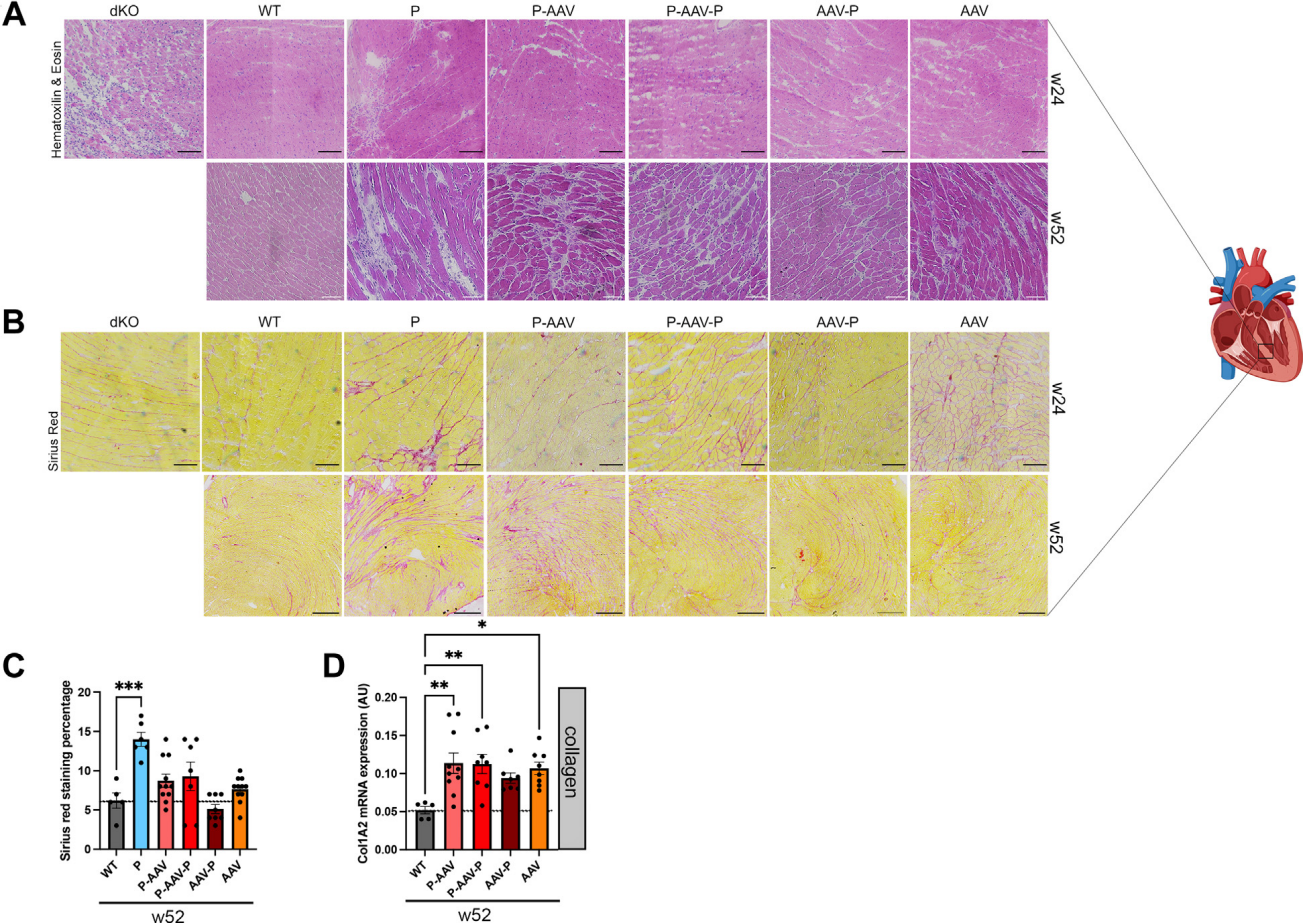
AAV-Micro-Dystrophin Treatment Induces Myocardial Inflammation Despite Low Fibrosis (A) Representative micrographs of hematoxylin & eosin–stained sections of hearts from 9-week-old double knockout (dKO) mice, wild-type (WT) mice, and dKO mice treated with peptide-conjugated phosphorodiamidate morpholino oligomer (PPMO) alone (P), PPMO+AAV-micro-dystrophin (P-AAV), PPMO+AAV-micro-dystrophin+PPMO (P-AAV-P), AAV-micro-dystrophin+PPMO (AAV-P), and AAV-micro-dystrophin alone (AAV) at weeks 24 and 52. dKO mice treated with PPMO alone were 38 to 50 weeks old. Scale bars: 100 μm. (B) Representative micrographs of Sirius red–stained sections of hearts from 9-week-old dKO mice, WT mice, and dKO mice treated with PPMO alone (P), PPMO+AAV-micro-dystrophin (P-AAV), PPMO+AAV-micro-dystrophin+PPMO (P-AAV-P), AAV-micro-dystrophin+PPMO (AAV-P), and AAV-micro-dystrophin alone (AAV) at weeks 24 and 52. dKO mice treated with PPMO alone were 38 to 50 weeks old. Scale bars: 100 μm. (C) Bar graphs showing fibrosis calculated as a percentage of the total area with QuPath software, from Sirius red–stained sections from 52-week-old mice. dKO mice treated with PPMO alone were 38 to 50 weeks old. Data are means ± SEM of at least 5 mice per group, and significance was determined by 1-way analysis of variance with Dunnett’s multiple comparison test to WT. (D) Bar graphs showing quantitative real-time polymerase chain reaction analysis of Col1A2 expression in the hearts of 52-week-old WT and dKO mice treated with PPMO alone (P), PPMO+AAV-micro-dystrophin (P-AAV), PPMO+AAV-micro-dystrophin+PPMO (P-AAV-P), AAV-micro-dystrophin+PPMO (AAV-P), and AAV-micro-dystrophin alone (AAV). dKO mice treated with PPMO alone were 38 to 50 weeks old. Data are means ± SEM for at least 5 mice per group, and significance was determined by 1-way analysis of variance (Dunnett’s multiple comparison with WT). ∗*P* < 0.05, ∗∗*P* < 0.01, ∗∗∗*P* < 0.001. Abbreviation as in Figure 1.

Despite normal cardiac function, the structure of cardiac muscle was altered in dKO mice treated with AAV-micro-dystrophin compared with untreated WT mice (Figure 5A). Sirius red staining showed no significant increased myocardial fibrosis in dKO mice treated with AAV-micro-dystrophin compared with WT mice (Figure 5B). The expression of *Col1a2*, a gene encoding collagen, was however significantly increased in dKO mice treated with AAV-micro-dystrophin compared with untreated WT mice (Figure 5C). Because RT-PCR is a more sensitive method compared with histologic staining, our dataset suggests an increased myocardial fibrosis. Hence, micro-dystrophin replacement therapy seems to halt the progression of myocardial fibrosis in a severe model of DMD without fully correcting it compared with a physiological condition. Histologic examination of hematoxylin and eosin–stained sections of cardiac muscle by a pathologist blinded to treatment showed mononuclear cell infiltration in most dKO mice treated with AAV-micro-dystrophin compared with dKO mice that were untreated and received PPMO only (Supplemental Table 4), suggesting cardiac inflammation.

**Figure 6:**
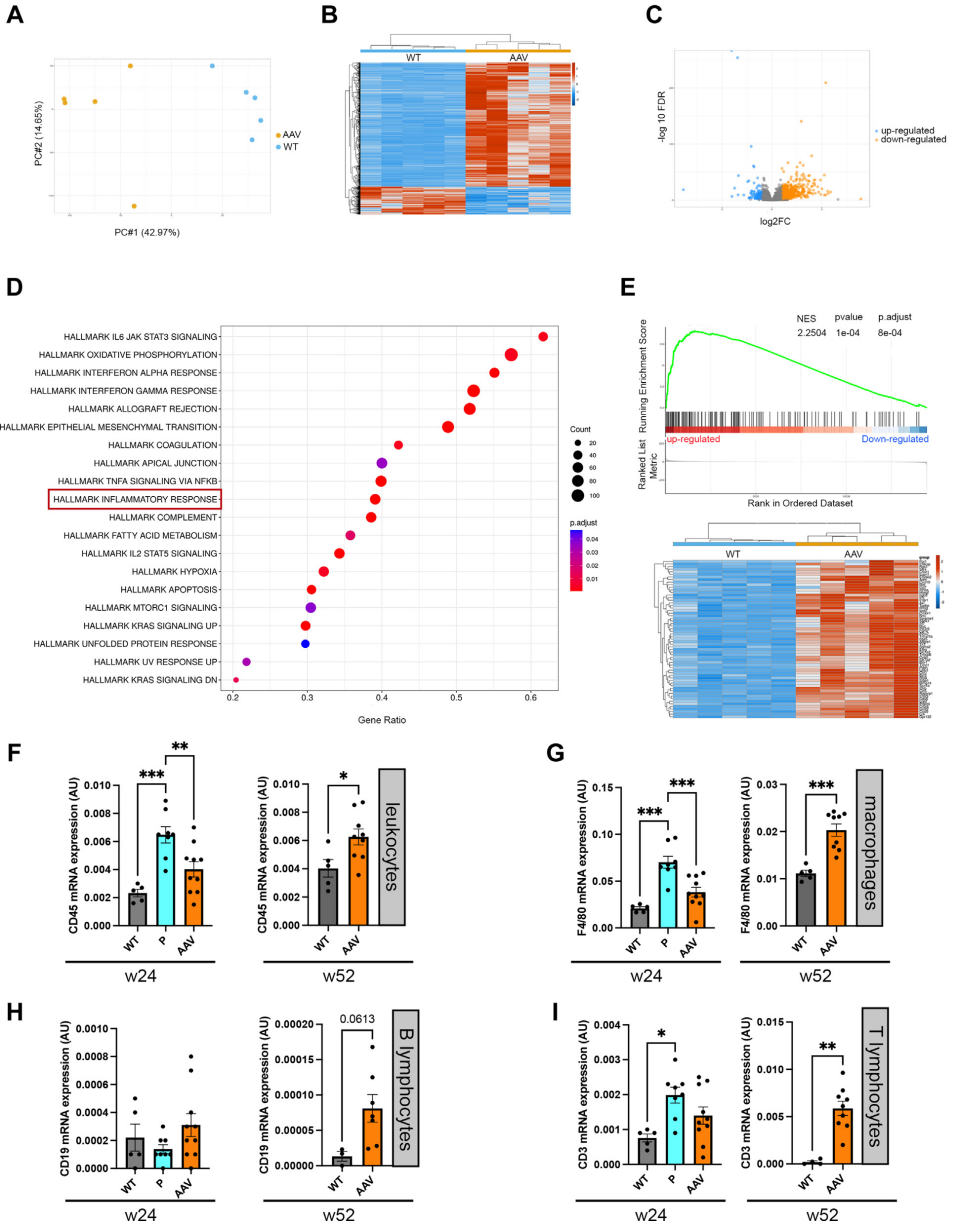
AAV-Micro-Dystrophin Treatment Induces Inflammatory Pathways Overexpression (A) Principle component analysis (PCA) mapped scatter plot: the global gene expression profiles of the heart from both 52-week-old wild-type (WT) mice and double knockout (dKO) AAV treated mice were analyzed by PCA. The figure represents the first 2 principal components of microarray analysis data (PC1, PC2) in X and Y, respectively, and demonstrated the expression profile of the 5 mice per group (WT blue, AAV orange). (B) Heat map analysis of microarray data showing hierarchical clustering of 1,129 differentially expressed probes between 52-week-old untreated WT and dKO AAV-treated hearts. Each group has 5 mice tested. Red or blue colors indicate differentially up- or down-regulated genes, respectively. (C) Volcano plot of differentially regulated genes between 52-week-old untreated WT and dKO AAV-treated hearts. The mean signals were background corrected and transformed to the log2 scale. Genes with at least 2-fold changes with *P* < 0.05 at the 95% confidence level were considered significant. (D) Enrichment analysis using hallmark gene sets collection. Data showed only pathways significantly regulated. (E) Gene set enrichment analysis plot (upper panel) and heatmap (lower panel) of hallmark inflammatory response gene expression. (F-I) Bar graphs showing quantitative real-time polymerase chain reaction analysis of CD45 (F), F4/80 (G), CD19 (H), and CD3 (I) expression in the hearts of 52-week-old WT mice and dKO mice treated with peptide-conjugated phosphorodiamidate morpholino oligomer (PPMO) (P), PPMO+AAV-micro-dystrophin (P-AAV), PPMO+AAV-micro-dystrophin+PPMO (P-AAV-P), AAV-micro-dystrophin+PPMO (AAV-P), and AAV-micro-dystrophin alone (AAV). dKO mice treated with PPMO alone were 38 to 50 weeks old. Data are means ± SEM for at least 5 mice per group, and significance was determined by 1-way analysis of variance with Dunnett’s multiple comparison test to WT. ∗*P* < 0.05, ∗∗*P* < 0.01, ∗∗∗*P* < 0.001. Abbreviation as in Figure 1.

To elucidate, at the molecular level, the cardiac pathogenesis induced by AAV-micro-dystrophin, we analyzed heart tissue collected from both 52-week-old WT and dKO AAV-treated mice using genome-wide microarray analysis. The 2 groups showed clear separation according to principal component analysis (Figure 6A) and a heat map constructed by unsupervised hierarchical clustering analysis (Figure 6B). We next used a supervised learning method to distinguish probe sets representing genes with significant differential expression in the hearts from the 2 groups of mice, revealing genes that were up- and down-regulated between the different groups (Figure 6C, Supplemental Table 5). Among the identified differentially expressed genes, the genes encoding inflammatory response components were significantly differentially expressed in the hearts of dKO mice treated with AAV-micro-dystrophin (Figures 6D and 6E, Supplemental Table 6). We confirmed RNA sequential data by quantitative RT-PCR and observed an increased *CD45* mRNA expression level in AAV-micro-dystrophin–treated animals compared with WT mice (Figure 6F). We also studied the expression of *F4/80* mRNA, a major macrophage marker, and showed a significant increase in expression in AAV-micro-dystrophin–treated animals compared with WT mice (Figure 6G).

To better characterize these macrophage populations, we investigated the expression of inflammatory and anti-inflammatory cytokines as well as macrophage markers (Supplemental Figure 8). We found increased mRNA levels of inflammatory cytokines (tumor necrosis factor-α, interleukin [IL]6) and CD80 marker (Supplemental Figure 8A), whereas IL10 was the only anti-inflammatory cytokine increased in AAV-treated dKO mice (Supplemental Figure 8A). It should be noted that IL10 is described to be both immunostimulatory and anti-inflammatory depending on its expression level.^34^ Because of the complexity of macrophage biology, these conflicting signals frequently overlap in dystrophic cardiomyopathy. We also looked for T-lymphocyte marker expression (Figures 6H and 6I) and showed an increase in *CD3* mRNA (Figure 6I). Furthermore, we reported that micro-dystrophin mRNA expression correlated with inflammatory markers (*CD45* and *F4/80*) (Supplemental Figure 9A), remodeling marker (*Nppa*) (Supplemental Figure 9B), and fibrotic marker (*Col1a2*) (Supplemental Figure 9C) mRNA. However, we did not observe a significant correlation with either connexin 43 or α-actin mRNA expressions (Supplemental Figure 9D).

**Figure 7:**
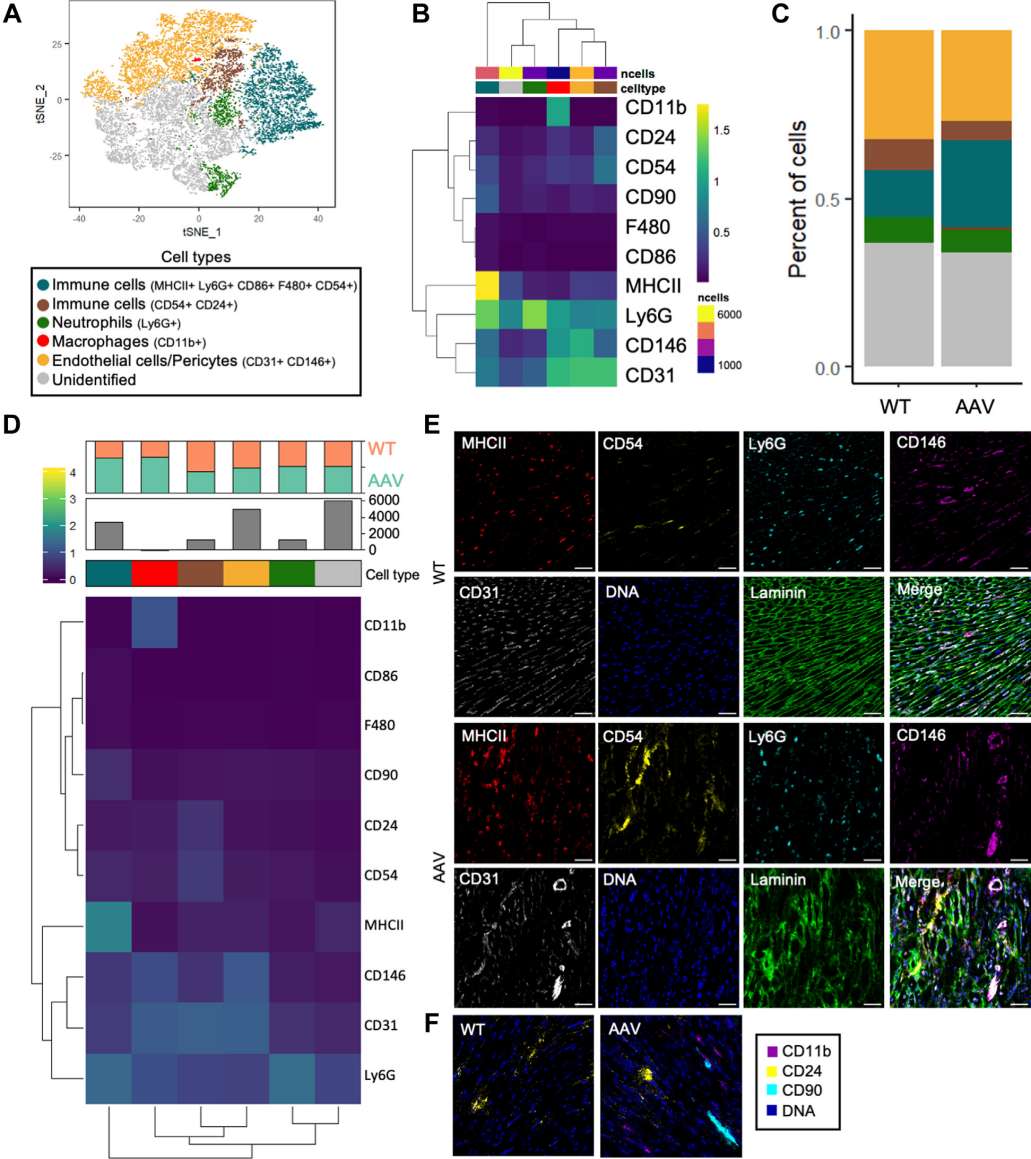
AAV-Micro-Dystrophin Treatment Induces Over-Representation Of Immune Cells (A) T-distributed stochastic neighbor embedding (t-SNE) of the imaging mass cytometry (IMC) dataset. (B) Heat map of average marker expression for each phenotypic cell cluster. Colored bars indicate clusters corresponding to specific cell types as indicated in A. (C) Percentages of different phenotypic cell clusters in untreated wild-type (WT) and AAV-micro-dystrophin–treated double knockout (dKO) heart septum. (D) Heat map of average marker expression for each phenotypic cell cluster. Colored bars indicate clusters corresponding to specific cell types, number of cells in the cluster, and indication of the most prevalent group of mice. (E) IMC images of 52-week-old untreated WT and AAV-micro-dystrophin–treated dKO mice heart septum stained for MHCII (red), CD54 (yellow), Ly6G (cyan), CD146 (magenta), CD31 (gray), DNA (blue), and laminin (green). (F) IMC images of 52-week-old untreated WT and AAV-micro-dystrophin treated dKO mice heart septum stained for CD11b (magenta), CD24 (yellow), CD90 (cyan), and DNA (blue). Abbreviation as in Figure 1.

To gain a deeper insight into cellular phenotype observed in hearts from dKO mice treated with AAV-micro-dystrophin, we next assessed their cardiac tissue using imaging mass cytometry, a mass spectroscopic technology that allows high-resolution, multidimensional characterization of cardiac cell surface and intracellular proteins at a single cell level, using heavy metal-tagged antibodies. We identified 9 different cellular clusters with distinct expression profiles, with respect to their intrinsic markers (Figure 7). We showed that inflammatory cells were over-represented (B cells, macrophages, granulocytes) in hearts from dKO mice treated with AAV-micro-dystrophin compared with untreated WT mice (Figure 7). We also confirmed these observations by immunofluorescence on MHCII marker (B cells) and CD54 (macrophages, monocytes), Ly6G (neutrophils), F4/80 (macrophages), and CD86 markers (monocytes, B-lymphocytes activation) (Figure 7). Altogether, these data suggest that AAV-micro-dystrophin triggers inflammation in cardiac muscle of dKO-treated mice, but this is not associated with functional cardiac declines.

**Figure 8:**
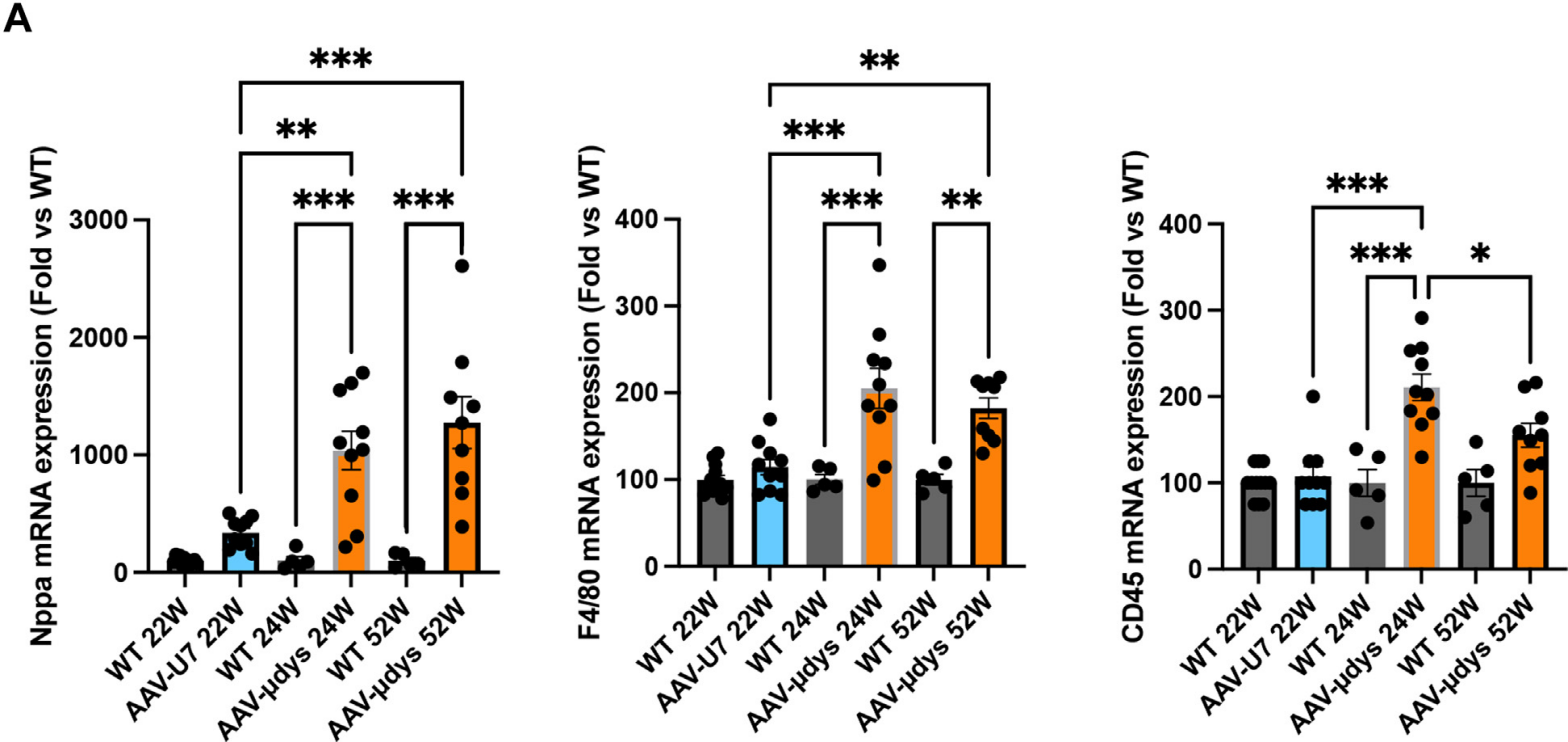
Cardiac Phenotype Arises From AAV-Micro-Dystrophin Overexpression (A) Bar graphs showing quantitative real-time polymerase chain reaction analysis of Nppa (left panel), F4/80 (midle panel), and CD45 (right panel) expression in the hearts from 22-week-old wild-type (WT) mice and AAV-U7–treated double knockout (dKO) mice, from 24-week-old WT mice and AAV-micro-dystrophin–treated dKO mice, and from 52-week-old WT mice and AAV-micro-dystrophin–treated dKO mice. The mice treated with AAV U7, as reported by Forand et al,^14^ represent 30% of the initial population. The remaining 70% succumbed to respiratory arrest. Data are mean ± SEM for at least 5 mice per group, and significance was determined by 1-way analysis of variance (with Tukey’s multiple comparison test). ∗*P* < 0.05, ∗∗*P* < 0.01, ∗∗∗*P* < 0.001. Abbreviation as in Figure 1.

In view of our results, the question has been raised about whether AAV or micro-dystrophin transgene expression was responsible for the cardiac inflammatory state observed. In our previous study, we used the same AAV serotype (AAV2/9) for U7-exon skipping strategy (AAV-U7) that was injected systemically at similar dose that the present study.^14^ We found increased mRNA expression of inflammatory (*F4/80*, *CD45*) and cardiac remodeling (*Nppa*) markers in AAV-micro-dystrophin–treated mice at 24 weeks of age compared with the AAV-U7–treated mice at the same age (Figure 8A). Moreover, mice treated with a combination PPMO-AAV-U7 approach survived until 52 weeks of age without showing septum thickening or inflammation. These data strongly suggest that cardiac side effects observed in the present study in the dKO mouse model using AAV-micro-dystrophin arise from transgene overexpression and not from immune response against AAV capsid. A strong argument supporting this claim comes from the kinetic of cellular immune response against the AAV capsid, which is known to occur early after AAV injection, within 4 to 8 weeks,^35^^,^^36^ and is usually associated with the loss of transgene expression resulting in decreased therapeutic efficacy,^37^, ^38^, ^39^, ^40^ which is not what we observed in the present study.

**Figure 9:**
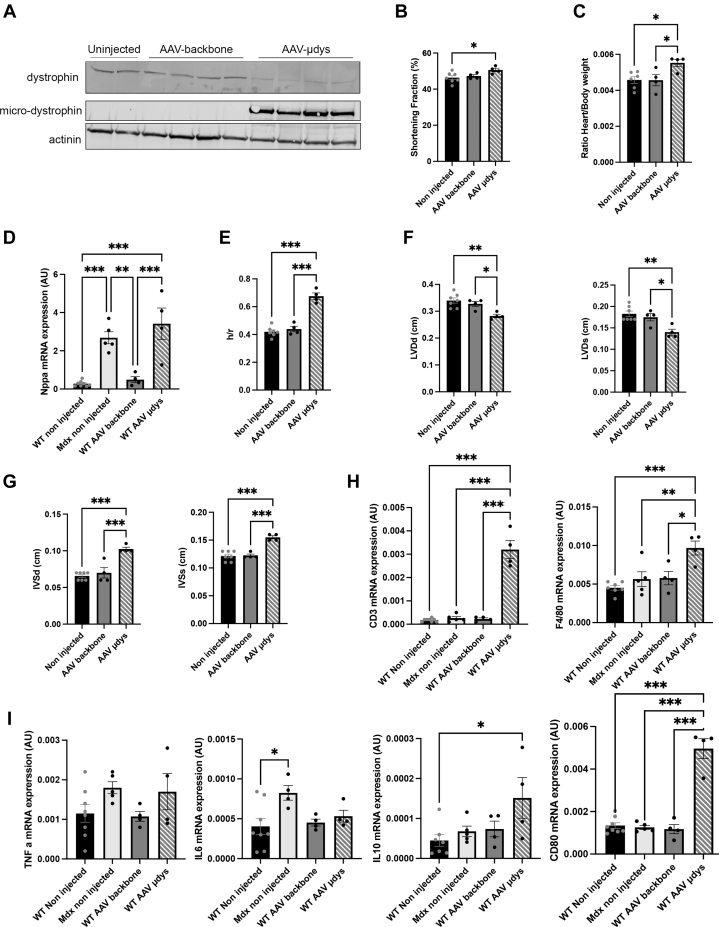
AAV-Micro-Dystrophin Overexpression Alters Cardiac Structure and Function in WT Mice (A) Representative immunoblots showing dystrophin and micro-dystrophin expression in hearts of 24-week-old WT mice treated with AAV-micro-dystrophin. α-Actinin was shown as a loading control. (B) Bar graphs showing the fractional shortening in 24-week-old WT noninjected mince and mice treated with AAV-backbone and AAV-micro-dystrophin (AAV-μdys). (C) Bar graphs showing the heart weight-to-body weight ratio in 24-week-old WT noninjected mice and mice treated with AAV-backbone, and AAV-micro-dystrophin (AAV-μdys). (D) Bar graphs showing real-time polymerase chain reaction analysis of *Nppa* in 24-week-old WT and mdx noninjected mice and WT mice treated with AAV-backbone and AAV-micro-dystrophin (AAV-μdys). (E) Bar graphs showing the h/r ratio in 24-week-old WT noninjected mice and mice treated with AAV-backbone and AAV-micro-dystrophin (AAV-μdys). (F) Bar graphs showing the left ventricular posterior wall thickness in diastole (LVPWd) and in systole (LVPWs) in 24-week-old WT noninjected mice and mice treated with AAV-backbone and AAV-micro-dystrophin (AAV-μdys). (G) Bar graphs showing the intraventricular end-diastolic thickness (IVSd) and end-systolic thickness (IVSs) in 24-week-old WT noninjected mice and mice treated with AAV-backbone and AAV-micro-dystrophin (AAV-μdys). (H) Bar graphs showing real-time polymerase chain reaction analysis of CD3 (T-lymphocytes) and F4/80 (macrophages) in 24-week-old WT and mdx noninjected mice and WT mice treated with AAV-backbone and AAV-micro-dystrophin (AAV-μdys). (I) Bar graphs showing real-time polymerase chain reaction analysis TNF-α, IL6, IL0, and CD80 in 24-week-old WT and mdx noninjected mice and WT mice treated with AAV-backbone and AAV-micro-dystrophin (AAV-μdys). (B-I) Data are means ± SEM of at least 4 mice per group. Significance was determined by 1-way analysis of variance when data were normally distributed or Kruskal-Wallis when they were not normally distributed. ∗*P* < 0.05, ∗∗*P* < 0.01, ∗∗∗*P* < 0.001. Abbreviation as in Figure 1.

Another question raised from our data is whether this inflammatory context was a consequence of AAV-micro-dystrophin treatment or mice dystrophic phenotype. We thus injected AAV-backbone or AAV-micro-dystrophin in WT mice. We first confirmed that both micro-dystrophin and dystrophin proteins were expressed in injected animals (Figure 9A). The cardiac function was next assessed by echocardiography (Figure 9B, Supplemental Table 6) and ECG (Supplemental Table 7). We found an alteration of cardiac function (Figure 9B, Supplemental Table 7) and structure only in AAV-micro-dystrophin–injected WT mice. Indeed, the ratio of heart-to-body weight was increased in the AAV-micro-dystrophin–treated mice (Figure 9C). This was ensuing, and the increased *Nppa* mRNA level (Figure 9D) and increased h/r ratio (Figure 9E) suggested cardiac remodeling. This was confirmed by a significant decreased left ventricular diameter (Figure 9F, Supplemental Table 7) and a thickening of the intra-ventricular septum (Figure 9G, Supplemental Table 7). Furthermore, histology analysis revealed inflammatory infiltration in AAV-micro-dystrophin–treated mice (Supplemental Figure 10). To determine whether the loss of full-length dystrophin and the expression of a micro-dystrophin could trigger an immune response, we examined inflammation in hearts from mdx mice (a well-known mouse model for DMD) and in WT mice treated either with AAV-micro-dystrophin or AAV-backbone. RT-PCR analysis showed increased macrophage and T lymphocyte markers in AAV-micro-dystrophin–treated mice (Figure 9H). Moreover, similar to dKO-treated mice, we reported an inflammatory environment (increased IL10 and CD80 mRNA levels) in AAV-micro-dystrophin–treated WT mice (Figure 9I). Taken together, these data suggest that micro-dystrophin overexpression triggers cardiac inflammation, even though the cardiac function of the dKO mice was significantly improved over time with the micro-dystrophin gene replacement therapy. The development of this inflammatory response could result from accumulation of micro-dystrophin.

We then study cellular processes involved in degradation of accumulated proteins. We showed an increased p62 cardiac expression, reporter of autophagy activity (Supplemental Figures 11A to 11C), and an accumulation of ubiquitinated proteins (Supplemental Figures 11B to 11D) in AAV-micro-dystrophin–treated WT and dKO mice. Moreover, RNA sequential data showed increased expression of the gene network involved in the unfolding protein response in the dKO AAV-micro-dystrophin–treated mice compared with untreated WT mice (Figure 6D). Altogether, our results suggest a defect in protein degradation processes, consistent with high levels of micro-dystrophin cardiac expression in AAV-micro-dystrophin–injected animals. This deserves greater attention for future studies.

## Discussion

Four clinical trials based on micro-dystrophin gene therapy are currently underway^10^, ^11^, ^12^ (NCT03368742 Solid Biosciences; NCT03375164, NCT03769116, NCT04626674, and NCT05096221 Sarepta Therapeutics; NCT03362502, NCT04281485, and NCT05429372 Pfizer, EudraCT Number 2020-002093-27 Genethon; and NCT05693142 Regenxbio), and very recently Sarepta Therapeutics announced US Food and Drug Administration approval of ELEVIDYS, the first gene therapy to treat DMD. Although it is obvious that these are promising therapeutic approaches, recent reports described limitations because of startling unwanted effects in both skeletal and cardiac muscles of treated DMD patients.^9^^,^^12^^,^^41^ Recently, the fatal outcome of a young DMD patient enrolled in a phase 2 clinical trial (NCT05429372) support these concerns. Hence, we studied the long-term effect of an AAV micro-dystrophin in combination, or not, with PPMO in a severe mouse model of DMD. We showed that a single injection in young dKO mice induced the expression of micro-dystrophin in the heart and prevented the development of dilated cardiomyopathy. We described that this therapy leads to cardiac structure alteration characterized by intraventricular septum thickening and myocardial inflammation (Figure 8B). Given that the dKO mice die at around 9 weeks of age from respiratory failure before the development of advanced cardiomyopathy, the best available control for longitudinal studies was therefore the group of mice treated with PPMO alone. These recapitulate the progression of DMD cardiomyopathy because this treatment is weakly effective in the heart,^28^^,^^29^ whereas it allows the correct rescue of dystrophin in the diaphragm and skeletal muscle.^14^ Even though only 2 mice treated with PPMO alone survived until 48 weeks, to overcome this limitation and exclude an impact of the dystrophic phenotype from our observations, we next compared WT mice injected with AAV-backbone or AAV-micro-dystrophin. We reported similar cardiac remodeling compared with AAV micro-dystrophin–treated dKO mice. These results revealed, for the first time, that the micro-dystrophin gene overexpression *per se* may lead to cardiac alterations over time. It would be insightful to compare our findings with functional cardiac measurements from DMD patients currently in clinical trials for micro-dystrophin gene therapy.

We previously demonstrated a striking benefit of combining exon skipping mediated by AAV-U7 with PPMO therapy on striated muscles with significant extent of survival of dKO-treated mice, opening novel therapeutic perspectives for patients.^14^ However, even if the combined treatment significantly mitigated both the heart and respiratory dysfunctions that are fatal for the DMD patients, our data strongly suggested that the dKO mice treated with the combined therapy died after 1 year from respiratory failure because of the loss of the PPMO therapeutic effect.^14^ These results revealed that further preclinical optimizations for this therapeutic approach are required. Here, we studied the long-term effect of an AAV micro-dystrophin combined with pre- and/or post-PPMO treatment in a severe mouse model of DMD. The AAV-micro-dystrophin treatment and the different combined therapies restored normal function of all striated muscles with a profound improvement of survival. Nevertheless, although our results confirmed that PPMO pretreatment is beneficial for the diaphragm, as previously reported,^14^ we also revealed that when used as combinatory post-treatment with AAV gene therapy, it seems to be associated with increased inflammation and fibrosis. One hypothesis is that the onset of fibrosis occurring during the time of AAV-only treatment could alter the elasticity of the diaphragm^42^ and that this phenomenon would then be exacerbated by the dystrophin expression induced by PPMO post-treatment that increases membrane actin stiffness.^43^ Because no alteration of function or inflammation could be detected in dKO mice receiving the early PPMO pretreatment, this suggests that it was beneficial to preserve the diaphragm to injury before AAV-micro-dystrophin and PPMO post-treatment injections.^44^

Because of an absence of a strong preclinical dystrophic mouse model that recapitulate human DMD cardiomyopathy progression, it is difficult to study the cardiac pathologic process.^26^^,^^45^, ^46^, ^47^, ^48^, ^49^ Micro-dystrophin gene replacement therapy has been shown to be beneficial for cardiac function in recent studies^45^^,^^50^ using the Fiona/dKO mice and the DMD rat model.^7^ These studies claimed benefits of micro-dystrophin therapy on myocardial inflammation and cardiac function in the long term.^45^^,^^50^ However, the Fiona/dKO mice is characterized by a stable cardiac function over time that is not significantly divergent from Fiona/dKO mice treated with micro-dystrophin.^35^ Similarly, the intraventricular septum was not different in untreated and treated Fiona/dKO mice at 18 months of age when the authors studied the long-term benefit of the therapeutic intervention. Therefore, it is difficult to assess the effect of micro-dystrophin gene replacement therapy in this mouse model. The dKO mice closely phenocopies the dystrophic pathology in DMD, including progressive degeneration of the skeletal muscles and diaphragm muscle and a cardiomyopathy associated with premature death. Hence, this model can be an effective approach for studying the dystrophic pathology and evaluating the efficacy of gene replacement therapy within a reasonable time frame along the disease progression.^17^ Nevertheless, the lack of utrophin in the dKO model could exacerbate the development of the pathology, which may not be observed in DMD patients. We report here that although AAV-micro-dystrophin–treated dKO mice have a significant improvement in their cardiac function, they developed cardiac side effects characterized by septum and posterior wall thickening, which were not observed in the untreated or PPMO-treated dKO mice. It should be noted that such a thickening of the walls was also reported in the GRMD dog treated with AAV-micro-dystrophin gene therapy, for which the cardiac function was normal.^51^ We also observed such cardiac modifications in WT mice injected with AAV-micro-dystrophin, which are therefore independent of the dystrophic phenotype. These clearly showed a remodeling of the cardiac tissue associated with micro-dystrophin. We hereby claim that features of cardiac inflammation observed in AAV-micro-dystrophin–treated dKO mice were a signature of the overexpression of micro-dystrophin protein as opposed to the normal progression of the disease (Figure 8B).

Cardiac side effects have been reported in patients using different versions of micro-dystrophin,^12^^,^^13^^,^^52^ suggesting that the dose of micro-dystrophin triggers the cardiac defects. Studies have shown that a protein level threshold exists beyond which a cardiotoxicity appears, as shown for mini-dystrophin that is cardiotoxic at 100-fold but not at 50-fold overexpression.^53^ In the design of micro-dystrophin protocols, an effort was made to optimize cardiac expression with a striated muscle specific promoter and by using the AAV serotype to efficiently transduce cardiomyocytes. However, several studies have reported cardiotoxicity in rodents when a foreign DNA sequence has been overexpressed.^54^, ^55^, ^56^, ^57^, ^58^ Moreover, cardiac remodeling occurred earlier in WT mice already expressing dystrophin. Another line of reasoning supporting that micro-dystrophin could be the cause of the cardiac inflammation comes from the fact that inflammatory infiltrates were not reported in dKO mice treated with U7 gene therapy using the same AAV vector. Although an early immune response directed against micro-dystrophin in mdx mice^59^ or DMD patients^13^ has been shown, we observed a late, progressive cardiac inflammatory response that could result from accumulation of micro-dystrophin. This is supported by our results showing abnormal regulation of protein degradation processes that could initiate inflammation.^60^^,^^61^ All these molecular pathways might be potential targets to prevent the cardiac inflammation. A wide panel of immune cell types (macrophages, leukocytes macrophages, dendritic cells, granulocytes, B and T cells, and natural killer cells) can be found in the cardiac tissue, contributing to immunosurveillance to protect against injuries and to maintain cardiac pump function. Clinicians have long appreciated that immune activation can negatively impact cardiac function.^60^ Targeting subsets of proinflammatory cell populations might offer opportunities for tailored therapy. Broad immune suppression has failed to demonstrate any benefit in multiple heart diseases.^61^ Therefore, the challenge is to identify targeted immune modulators that can specifically affect components of the immune response in DMD patients to diminish adverse remodeling. Targeted manipulation of the immune system to benefit the cardiac muscle is an exciting and promising field with great impact for future development of therapies.

Perspectives **COMPETENCY IN MEDICAL KNOWLEDGE:** DMD is a severe and progressive inherited muscular dystrophy caused by a loss of dystrophin, affecting both skeletal and cardiac muscles, affecting survival for patients. Gene transfer micro-dystrophin therapy improves muscle function and increased survival.

**TRANSLATIONAL OUTLOOK:** Our results underscore the long-term cardiac effect of this therapy, and we describe that it leads to cardiac structure alteration characterized by myocardial inflammation. Future research should analyze targeted immune modulators that can specifically affect components of the immune response in DMD patients treated with micro-dystrophin therapy for future development of therapies.

### Study Limitations

The main limitation of this study was the severity of the dKO mouse model. dKO mice typically die around 9 weeks of age due to respiratory failure, before the development of cardiomyopathy. As a result, we were unable to observe the pathophysiological progression of heart disease in these mice for comparison with treated mice. To address this, we used dKO mice treated with PPMO as controls. These mice replicate the progression of DMD cardiomyopathy, as the treatment has limited effectiveness in the heart, although it successfully rescues dystrophin in the diaphragm and skeletal muscle. However, only 2 PPMO-treated mice survived beyond 48 weeks. To mitigate this limitation, we treated WT mice with micro-dystrophin, ensuring that the observed phenotype was not influenced by the dystrophic background.

It is important to note that the preclinical cardiac effects of micro-dystrophin in DMD may be specific to the animal model used. Therefore, it would be important to compare our study with clinical data from DMD patients in clinical trials using micro-dystrophin.

## Conclusions

Despite the beneficial effects observed in DMD clinical trials, serious adverse effects on cardiac muscle have emerged in ongoing clinical studies.

Our study shows for the first time that micro-dystrophin gene replacement therapy in the dKO model can be associated with long-term cardiac alterations, and opens up perspectives for understanding the consequences of using this approach in DMD patients. Indeed, we argue that the features of cardiac inflammation observed in AAV-micro-dystrophin-treated dKO mice are a signature of micro-dystrophin protein overexpression rather than normal disease progression. These data pave the way for deciphering these events and preventing their side-effects, with a view to optimizing the treatment of DMD by micro-dystrophin gene therapy.

## Funding Support and Author Disclosures

The authors have reported that they have no relationships relevant to the contents of this paper to disclose.
