# Supplementary material for: Long-Term Dystrophin Replacement Therapy in Duchenne Muscular Dystrophy Causes Cardiac Inflammation
Source: JACC Basic Transl Sci. 2025 Mar 12;10(6):759–82. doi: 10.1016/j.jacbts.2024.12.015 (PMC12230499; doi:10.1016/j.jacbts.2024.12.015)
Supplement: Supplemental Material [file mmc1.pdf]

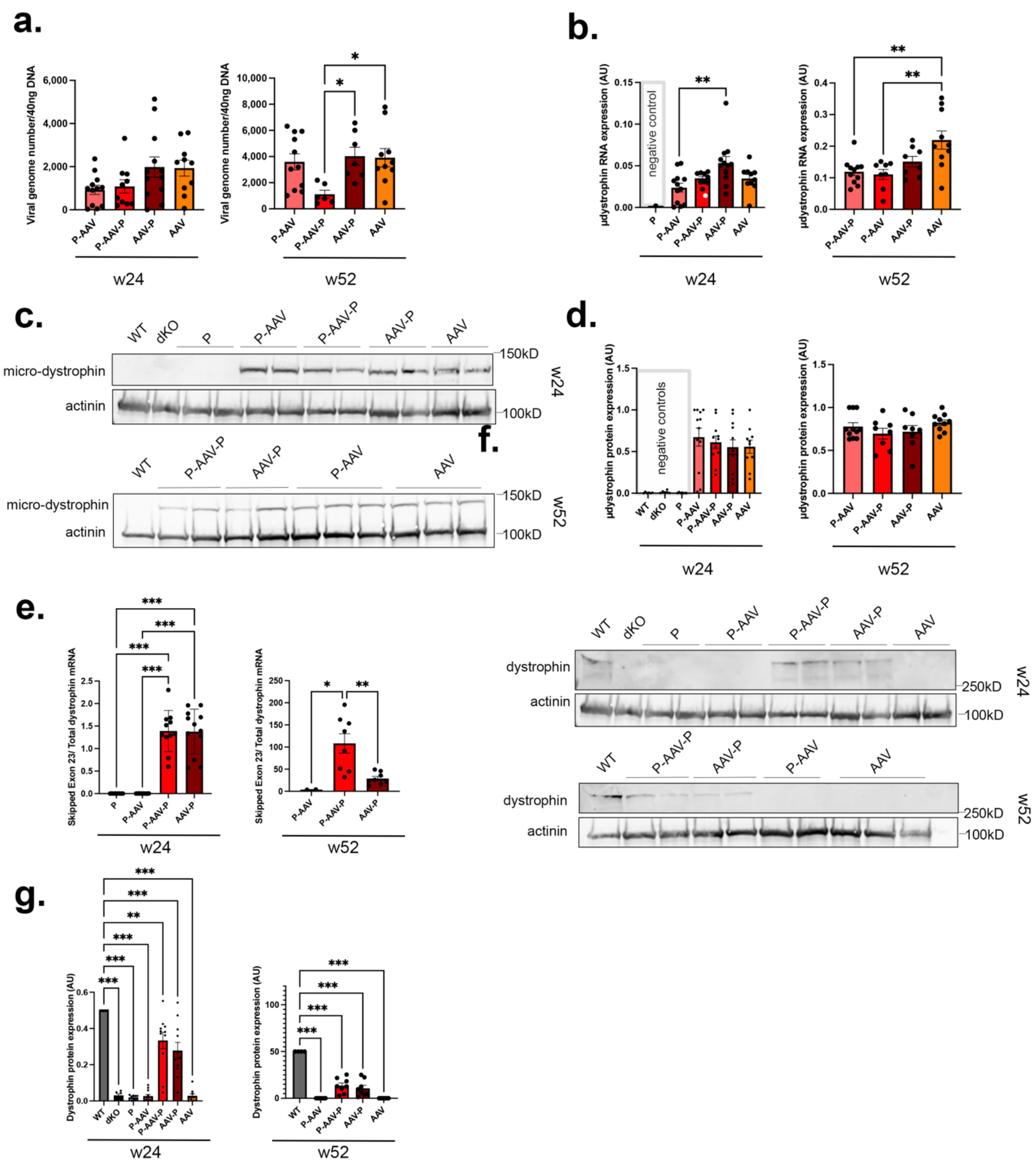

Supplementary Figure 1: (a) Quantification of viral genome number in diaphragm from 24W and 52W-old dKO treated with PPMO alone (P), PPMO+AAV-micro dystrophin (P-AAV), PPMO+AAV-micro dystrophin+PPMO (P-AAV-P), AAV-micro dystrophin+PPMO (AAV-P) and AAV-micro dystrophin alone (AAV). Each dot represents one mouse. Data are means ± SEM of at least 8 mice per group. Significance was determined by one-way ANOVA with Tukey's multiple comparison test. **(b)** Quantification of micro dystrophin gene expression in diaphragm from 24W and 52W-old dKO treated with PPMO alone (P), PPMO+AAV-micro dystrophin (P-AAV), PPMO+AAV-micro dystrophin+PPMO (P-AAV-P), AAV-micro

dystrophin+PPMO (AAV-P) and AAV-micro dystrophin alone (AAV). Each dot represents one mouse. Data are means ± SEM of at least 8 mice per group. Significance was determined by one-way ANOVA with Tukey's multiple comparison test. **(c)** Representative immunoblots showing micro dystrophin expression in diaphragm from 24W and 52W-old WT mice and dKO treated with PPMO alone (P), PPMO+AAV-micro dystrophin (P-AAV), PPMO+AAV-micro dystrophin+PPMO (P-AAV-P), AAV-micro dystrophin+PPMO (AAV-P) and AAV-micro dystrophin alone (AAV). α-actinin was shown as loading control. **(d)** Quantifications of micro dystrophin expression in diaphragm from 24W and 52W-old dKO treated with PPMO alone (P), PPMO+AAV-micro dystrophin (P-AAV), PPMO+AAV-micro dystrophin+PPMO (P-AAV-P), AAV-micro dystrophin+PPMO (AAV-P) and AAV-micro dystrophin alone (AAV). Graphs are means ± SEM of at least 8 mice per group. Significance was determined by one-way ANOVA with Tukey's multiple comparison test. **(e)** Quantification of exon 23 skipped dystrophin gene expression in diaphragm from 24W and 52W-old dKO treated with PPMO alone (P), PPMO+AAV-micro dystrophin (P-AAV), PPMO+AAV-micro dystrophin+PPMO (P-AAV-P), AAV-micro dystrophin+PPMO (AAV-P) and AAV-micro dystrophin alone (AAV). Each dot represents one mouse. Data are means ± SEM of at least 8 mice per group. Significance was determined by one-way ANOVA with Tukey's multiple comparison test. **(f)** Representative immunoblots showing skipped exon 23 dystrophin expression in diaphragm from 24W and 52W-old WT mice and dKO treated with PPMO alone (P), PPMO+AAV-micro dystrophin (P-AAV), PPMO+AAV-micro dystrophin+PPMO (P-AAV-P), AAV-micro dystrophin+PPMO (AAV-P) and AAV-micro dystrophin alone (AAV). α-actinin was shown as loading control. **(g)** Quantifications of skipped exon 23 dystrophin expression in diaphragm from 24W and 52W-old WT mice and dKO treated with PPMO alone (P), PPMO+AAV-micro dystrophin (P-AAV), PPMO+AAV-micro dystrophin+PPMO (P-AAV-P), AAV-micro dystrophin+PPMO (AAV-P) and AAV-micro dystrophin alone (AAV). Graphs are means ± SEM of at least 8 mice per group. Significance was determined by one-way ANOVA with Tukey's multiple comparison test. (*$p < 0.05$, **$p < 0.01$, ***$p < 0.001$).

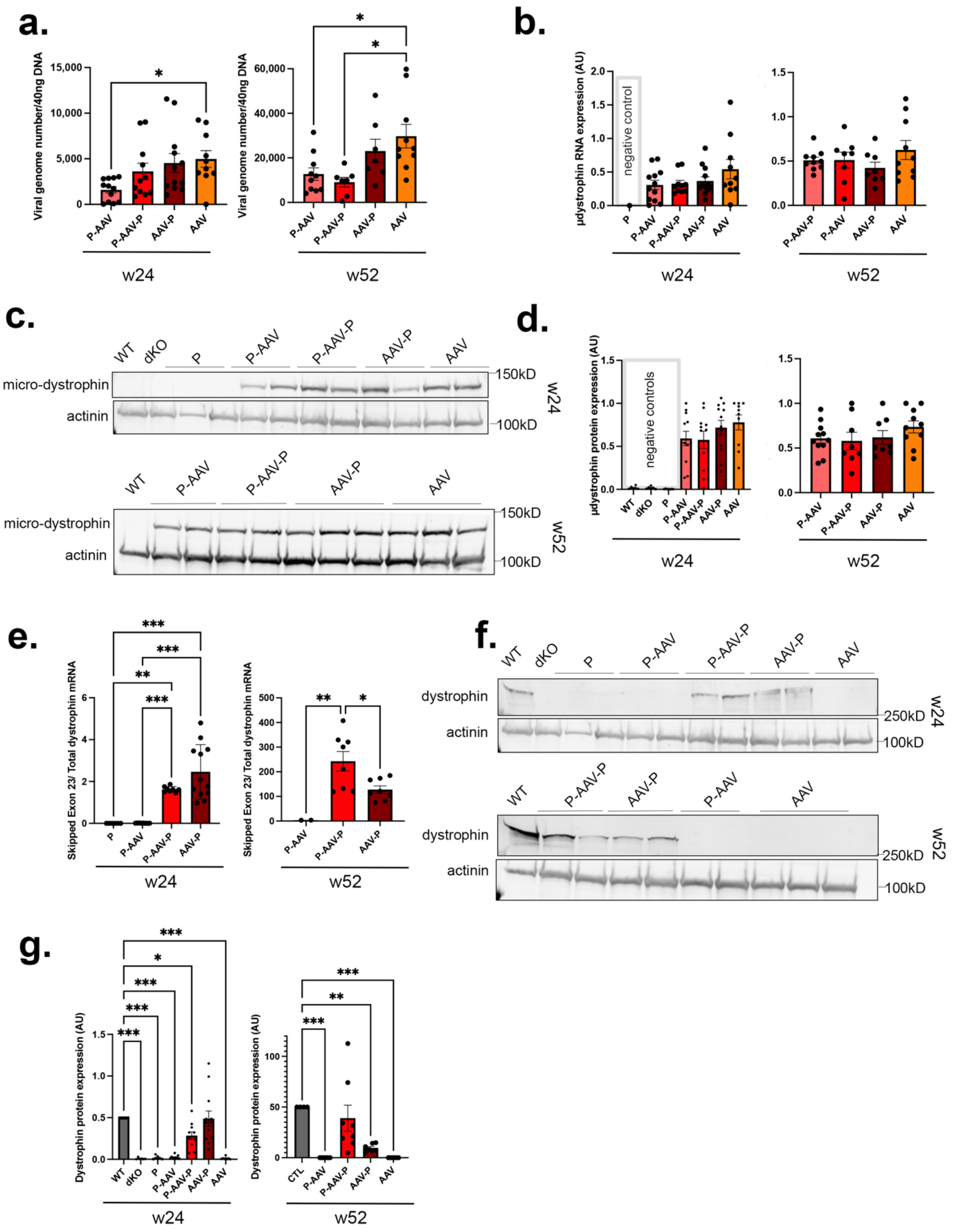

Supplementary Figure 2: (a) Quantification of viral genome number in TA from 24W and 52W-old dKO treated with PPMO alone (P), PPMO+AAV-micro dystrophin (P-AAV), PPMO+AAV-micro dystrophin+PPMO (P-AAV-P), AAV-micro dystrophin+PPMO (AAV-P) and AAV-micro dystrophin alone

(AAV). Each dot represents one mouse. Data are means ± SEM of at least 8 mice per group. Significance was determined by one-way ANOVA with Tukey's multiple comparison test. **(b)** Quantification of micro dystrophin gene expression in TA from 24W and 52W-old dKO treated with PPMO alone (P), PPMO+AAV-micro dystrophin (P-AAV), PPMO+AAV-micro dystrophin+PPMO (P-AAV-P), AAV-micro dystrophin+PPMO (AAV-P) and AAV-micro dystrophin alone (AAV). Each dot represents one mouse. Data are means ± SEM of at least 8 mice per group. Significance was determined by one-way ANOVA with Tukey's multiple comparison test. **(c)** Representative immunoblots showing micro dystrophin expression in TA from 24W and 52W-old WT mice and dKO treated with PPMO alone (P), PPMO+AAV-micro dystrophin (P-AAV), PPMO+AAV-micro dystrophin+PPMO (P-AAV-P), AAV-micro dystrophin+PPMO (AAV-P) and AAV-micro dystrophin alone (AAV). α-actinin was shown as loading control. **(d)** Quantifications of micro dystrophin expression in TA from 24W and 52W-old dKO treated with PPMO alone (P), PPMO+AAV-micro dystrophin (P-AAV), PPMO+AAV-micro dystrophin+PPMO (P-AAV-P), AAV-micro dystrophin+PPMO (AAV-P) and AAV-micro dystrophin alone (AAV). Graphs are means ± SEM of at least 8 mice per group. Significance was determined by one-way ANOVA with Tukey's multiple comparison test. **(e)** Quantification of exon 23 skipped dystrophin gene expression in TA from 24W and 52W-old dKO treated with PPMO alone (P), PPMO+AAV-micro dystrophin (P-AAV), PPMO+AAV-micro dystrophin+PPMO (P-AAV-P), AAV-micro dystrophin+PPMO (AAV-P) and AAV-micro dystrophin alone (AAV). Each dot represents one mouse. Data are means ± SEM of at least 8 mice per group. Significance was determined by one-way ANOVA with Tukey's multiple comparison test. **(f)** Representative immunoblots showing skipped exon 23 dystrophin expression in TA from 24W and 52W-old WT mice and dKO treated with PPMO alone (P), PPMO+AAV-micro dystrophin (P-AAV), PPMO+AAV-micro dystrophin+PPMO (P-AAV-P), AAV-micro dystrophin+PPMO (AAV-P) and AAV-micro dystrophin alone (AAV). α-actinin was shown as loading control. **(g)** Quantifications of skipped exon 23 dystrophin expression in TA from 24W and 52W-old WT mice and dKO treated with PPMO alone (P), PPMO+AAV-micro dystrophin (P-AAV), PPMO+AAV-micro dystrophin+PPMO (P-AAV-P), AAV-micro dystrophin+PPMO (AAV-P) and AAV-micro dystrophin alone (AAV). Graphs are means ± SEM of at least

8 mice per group. Significance was determined by one-way ANOVA with Tukey’s multiple comparison test. (*p < 0.05, **p < 0.01, ***p < 0.001).

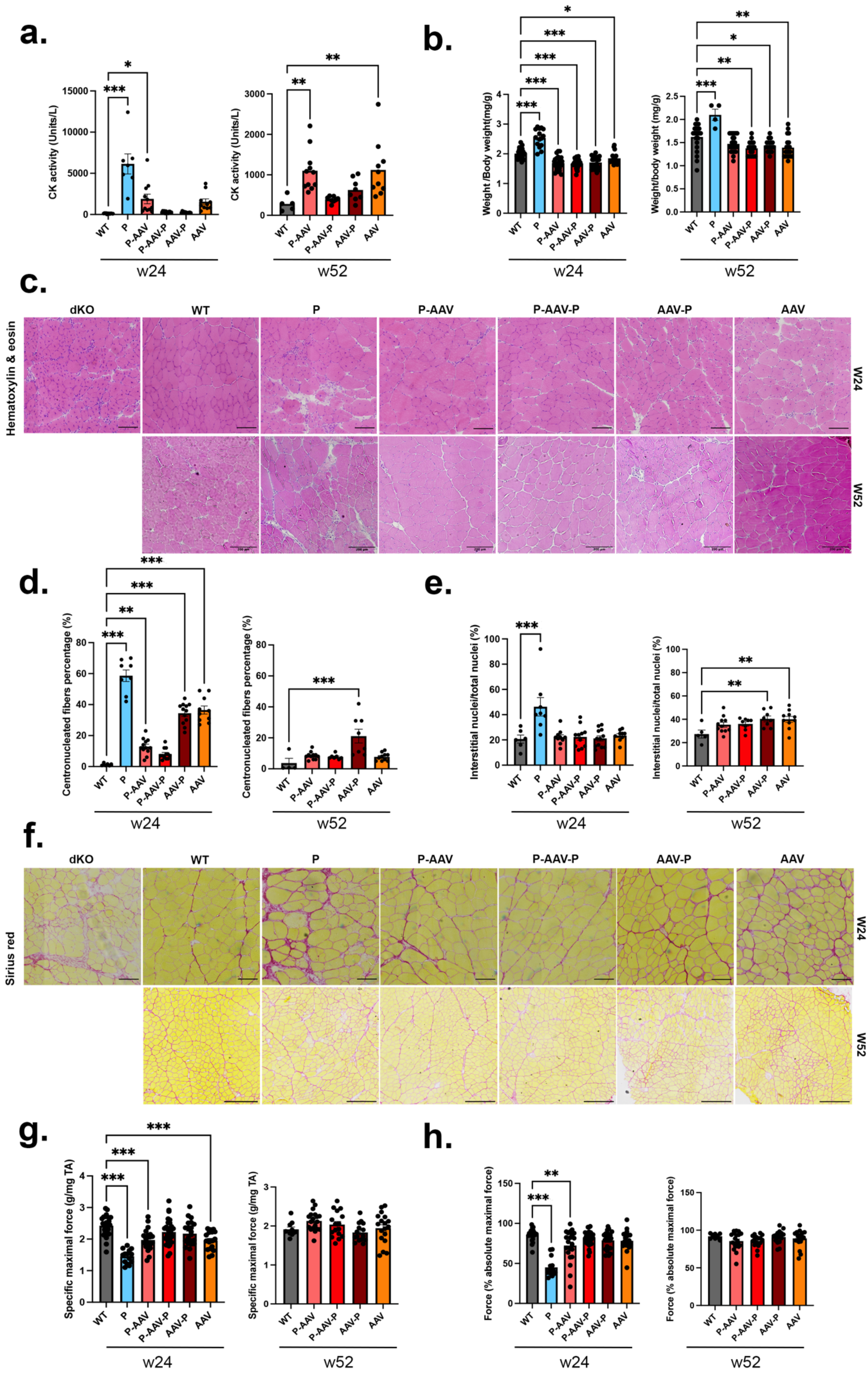

a.
b.
c.
d.
e.
f.
g.
h.
CK activity (Units/L)
Weight /Body weight(mg/g)
Weight/body weight (mg/g)
Hematoxylin & eosin
Sirius red
dKO
WT
P
P-AAV
P-AAV-P
AAV-P
AAV
W24
W52
Centronucleated fibers percentage (%)
Interstitial nuclei/total nuclei (%)
Specific maximal force (g/mg TA)
Force (% absolute maximal force)
w24
w52

Supplementary Figure 3: (a) Bar graphs showing the Creatine Kinase (CK) activity in 24W and 52W-old WT and dKO treated with PPMO alone (P), PPMO+AAV-micro dystrophin (P-AAV), PPMO+AAV-micro dystrophin+PPMO (P-AAV-P), AAV-micro dystrophin+PPMO (AAV-P) and AAV-micro dystrophin alone (AAV). Data are means ± SEM of at least 5 mice per group. Significance was determined by one-way ANOVA with Dunnett's multiple comparison to WT. **(b)** Ratios of TA to body weight from 24W and 52W-old dKO treated with PPMO alone (P), PPMO+AAV-micro dystrophin (P-AAV), PPMO+AAV-micro dystrophin+PPMO (P-AAV-P), AAV-micro dystrophin+PPMO (AAV-P) and AAV-micro dystrophin alone (AAV). Data are means ± SEM of at least 5 mice per group except for the PPMO group at 52W that is means ± SEM of 2 mice of 48W-old. Significance was determined by one-way ANOVA with Dunnett's multiple comparison to WT. **(c)** Representative micrographs of hematoxylin-eosin-stained sections of TA from 9W-old dKO mice, WT mice and dKO mice treated with PPMO alone (P), PPMO+AAV-micro dystrophin (P-AAV), PPMO+AAV-micro dystrophin+PPMO (P-AAV-P), AAV-micro dystrophin+PPMO (AAV-P) and AAV-micro dystrophin alone (AAV), at W24 and W52. Scale bars, 100 µm. (d) Bar graphs showing the centronucleated fiber percentage calculated with Qupath software in 24W and 52W-old WT and dKO treated with PPMO alone (P), PPMO+AAV-micro dystrophin (P-AAV), PPMO+AAV-micro dystrophin+PPMO (P-AAV-P), AAV-micro dystrophin+PPMO (AAV-P) and AAV-micro dystrophin alone (AAV). Data are means ± SEM of at least 5 mice per group. Significance was determined by one-way ANOVA with Dunnett's multiple comparison to WT. (e) Bar graphs showing the interstitial nuclei percentage calculated with Qupath software in 24W and 52W-old WT and dKO treated with PPMO alone (P), PPMO+AAV-micro dystrophin (P-AAV), PPMO+AAV-micro dystrophin+PPMO (P-AAV-P), AAV-micro dystrophin+PPMO (AAV-P) and AAV-micro dystrophin alone (AAV). Data are means ± SEM of at least 5 mice per group. Significance was determined by one-way ANOVA with Dunnett's multiple comparison to WT. **(f)** Representative micrographs of sirius red-stained sections of TA from 9W-old dKO mice, WT mice and dKO mice treated with PPMO alone (P), PPMO+AAV-micro dystrophin (P-AAV), PPMO+AAV-micro dystrophin+PPMO (P-AAV-P), AAV-micro dystrophin+PPMO (AAV-P) and AAV-micro dystrophin alone (AAV), at W24 and W52. Scale bars, 100 µm. **(g)** Specific maximal force and **(h)** force

drop resulting from the injury induced by 10 lengthening contractions of the TA for 52W-old WT and dKO mice treated PPMO alone (P), PPMO+AAV-micro dystrophin (P-AAV), PPMO+AAV-micro dystrophin+PPMO (P-AAV-P), AAV-micro dystrophin+PPMO (AAV-P) and AAV-micro dystrophin alone (AAV). Data are means ± SEM of at least 5 mice per group. Significance was determined by one-way ANOVA with Dunnett's multiple comparison to WT. (*$p < 0.05$, **$p < 0.01$, ***$p < 0.001$).

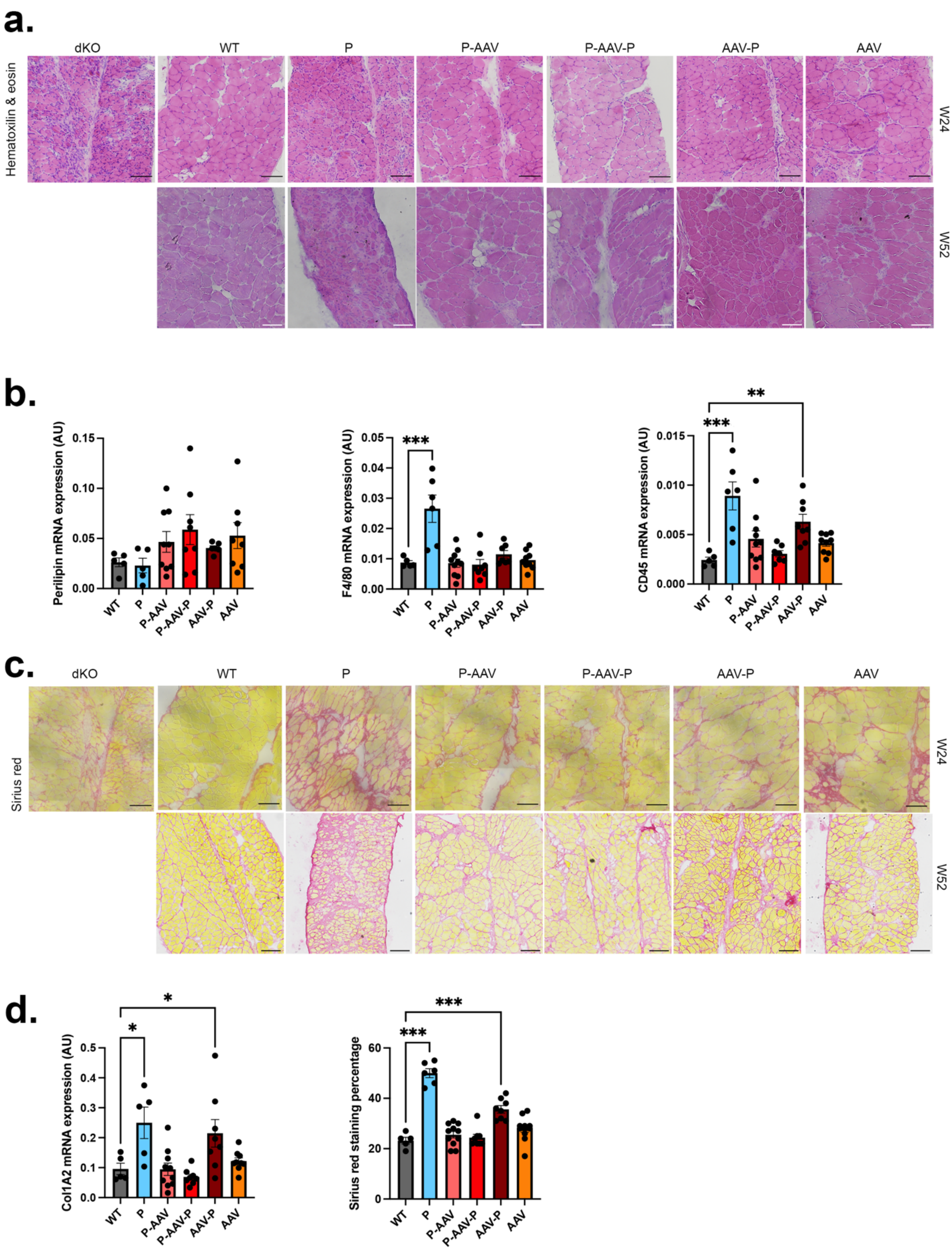

Supplementary Figure 4: (a) Representative micrographs of hematoxylin-eosin-stained sections of diaphragm from 9W-old dKO mice, WT mice and dKO mice treated with PPMO alone (P), PPMO+AAV-micro dystrophin (P-AAV), PPMO+AAV-micro dystrophin+PPMO (P-AAV-P), AAV-micro

dystrophin+PPMO (AAV-P) and AAV-micro dystrophin alone (AAV), at W24 and W52. Scale bars, 100 µm. **(b)** Bar graphs showing RT Q-PCR analysis of perilipin, F4/80 and CD45 expression in the diaphragms of 52W-old WT mice and dKO mice treated with PPMO (P), PPMO+AAV-micro dystrophin (P-AAV), PPMO+AAV-micro dystrophin+PPMO (P-AAV-P), AAV-micro dystrophin+PPMO (AAV-P) and AAV-micro dystrophin alone (AAV). dKO mice treated with PPMO alone are 38 to 50W-old. Data shown means ± SEM for at least 5 mice per group, and significance was determined by one-way ANOVA with Dunnett's multiple comparison to WT. **(c)** Representative micrographs of sirius red-stained sections of diaphragms from 9W-old dKO, WT and dKO mice treated with PPMO alone (P), PPMO+AAV-micro dystrophin (P-AAV), PPMO+AAV-micro dystrophin+PPMO (P-AAV-P), **(d)** Bar graphs showing RT Q-PCR analysis of Col1A2 expression (left panel) in the diaphragm of 52W-old WT and dKO mice treated with PPMO+AAV-micro dystrophin (P-AAV), PPMO+AAV-micro dystrophin+PPMO (P-AAV-P), AAV-micro dystrophin+PPMO (AAV-P) and AAV-micro dystrophin alone (AAV). Bar graphs showing fibrosis (right panel) calculated as a percentage of the total area with Qupath software, from sirius red-stained sections from 52-week-old mice. dKO mice treated with PPMO alone are 38 to 50W-old. The data shown are means ± SEM of at least five mice per group, and significance was determined by one-way ANOVA with Dunnett's multiple comparison to WT. (*$p < 0.05$, **$p < 0.01$, ***$p < 0.001$).

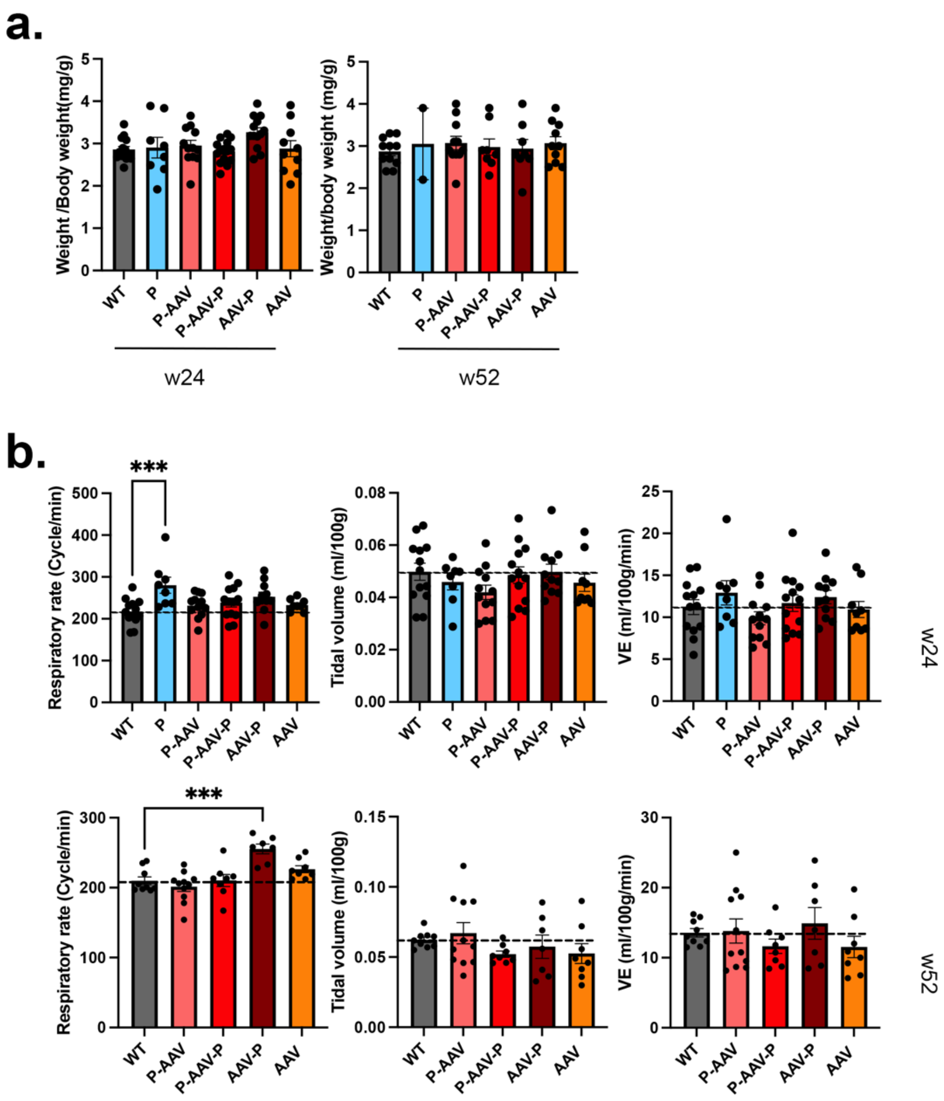

Supplementary Figure 5: (a) Ratios of diaphragm to body weight from 24W and 52W-old dKO treated with PPMO alone (P), PPMO+AAV-micro dystrophin (P-AAV), PPMO+AAV-micro dystrophin+PPMO (P-AAV-P), AAV-micro dystrophin+PPMO (AAV-P) and AAV-micro dystrophin alone (AAV). Data are means

± SEM of at least 8 mice per group except for the PPMO group at 52W that is means ± SEM of 2 mice of 48W-old. Significance was determined by one-way ANOVA with Dunnett's multiple comparison to WT. **(b)** Respiratory parameters from WT and dKO mice treated with PPMO alone (P), PPMO+AAV-micro dystrophin (P-AAV), PPMO+AAV-micro dystrophin+PPMO (P-AAV-P), AAV-micro dystrophin+PPMO (AAV-P) and AAV-micro dystrophin alone (AAV), at W24 (upper panel) and W52 (lower panel). fR, respiratory frequency; V_T, tidal volume; V_E, minute ventilation. The data shown are means ± SEM of at least 8 mice per group. Significance was determined by one-way ANOVA with Dunnett's multiple comparison to WT. (***$p < 0.001$).

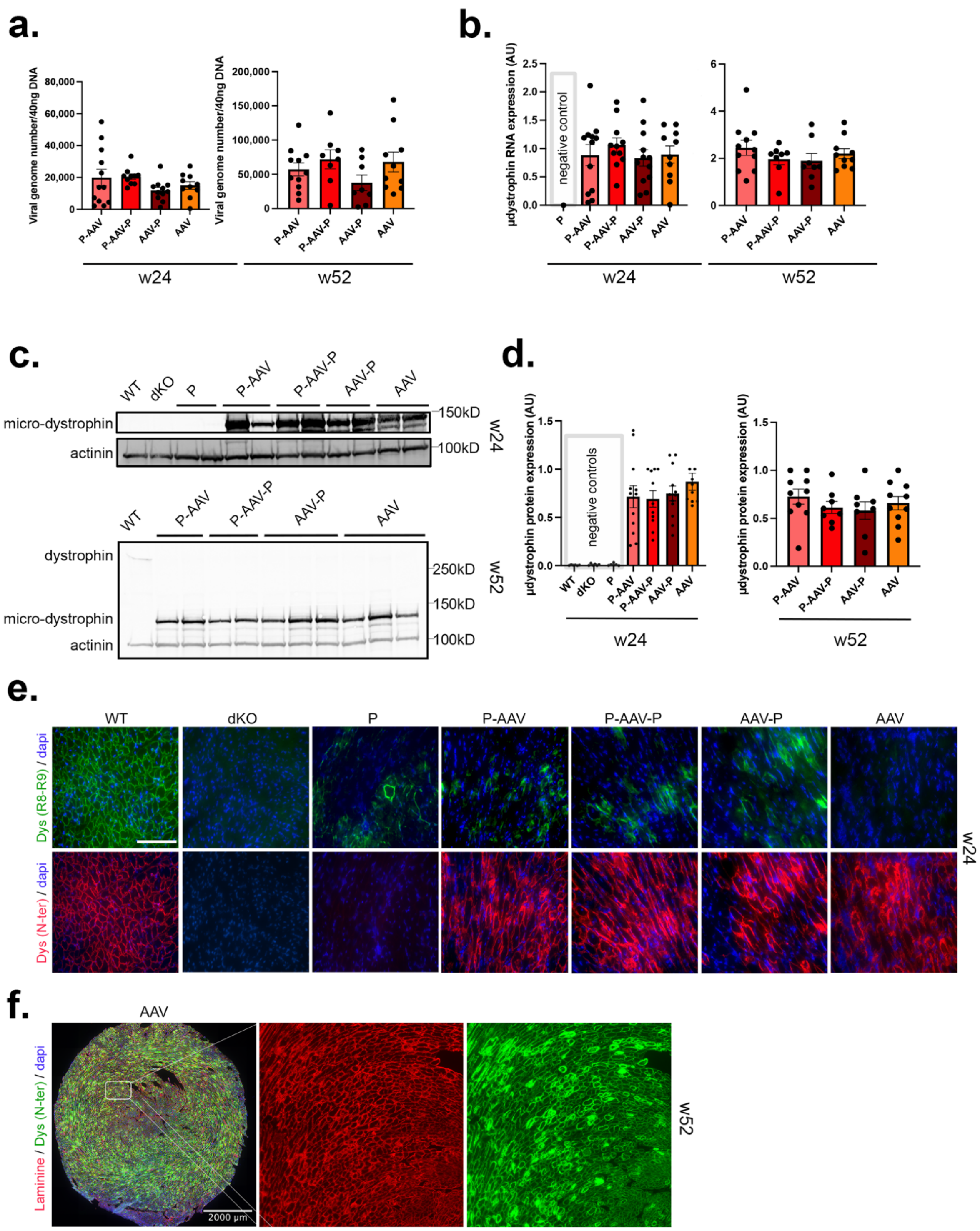

Supplementary Figure 6: (a) Quantification of viral genome number in heart from 24W and 52W-old dKO mice treated with PPMO alone (P), PPMO+AAV-micro dystrophin (P-AAV), PPMO+AAV-micro dystrophin+PPMO (P-AAV-P), AAV-micro dystrophin+PPMO (AAV-P) and AAV-micro dystrophin alone (AAV). Each dot represents one mouse. Data are means ± SEM of at least 8 mice per group. Significance

was determined by one-way ANOVA with multiple comparison (Tukey's test). **(b)** Quantification of micro dystrophin gene expression in heart from 24W and 52W-old dKO mice treated with PPMO alone (P), PPMO+AAV-micro dystrophin (P-AAV), PPMO+AAV-micro dystrophin+PPMO (P-AAV-P), AAV-micro dystrophin+PPMO (AAV-P) and AAV-micro dystrophin alone (AAV). Each dot represents one mouse. Data are means $\pm$ SEM of at least 8 mice per group. Significance was determined by one-way ANOVA with multiple comparison (Tukey's test). **(c)** Representative immunoblots showing micro dystrophin expression in heart from 24W and full-length dystrophin and micro-dystrophin in 52W-old WT and dKO mice treated with PPMO alone (P), PPMO+AAV-micro dystrophin (P-AAV), PPMO+AAV-micro dystrophin+PPMO (P-AAV-P), AAV-micro dystrophin+PPMO (AAV-P) and AAV-micro dystrophin alone (AAV). α-actinin was shown as loading control. **(d)** Quantifications of micro dystrophin expression in heart from 24W and 52W-old dKO mice treated with PPMO alone (P), PPMO+AAV-micro dystrophin (P-AAV), PPMO+AAV-micro dystrophin+PPMO (P-AAV-P), AAV-micro dystrophin+PPMO (AAV-P) and AAV-micro dystrophin alone (AAV). Graphs are means $\pm$ SEM of at least 8 mice per group. Significance was determined by one-way ANOVA with multiple comparison (Tukey's test). **(e)** Micrographs showing dystrophin and micro dystrophin labelling in heart from 24W-old dKO treated with PPMO alone (P), PPMO+AAV-micro dystrophin (P-AAV), PPMO+AAV-micro dystrophin+PPMO (P-AAV-P), AAV-micro dystrophin+PPMO (AAV-P) and AAV-micro dystrophin alone (AAV). Upper panel, antibody recognize dystrophin only (green), lower panel, antibody recognize both dsytrophin and micro dystrophin (red). Scale bar=100µm. **(f)** Micrographs showing laminin (red) and micro-dystrophin (green) labelling in heart from 52W-old dKO treated with AAV.

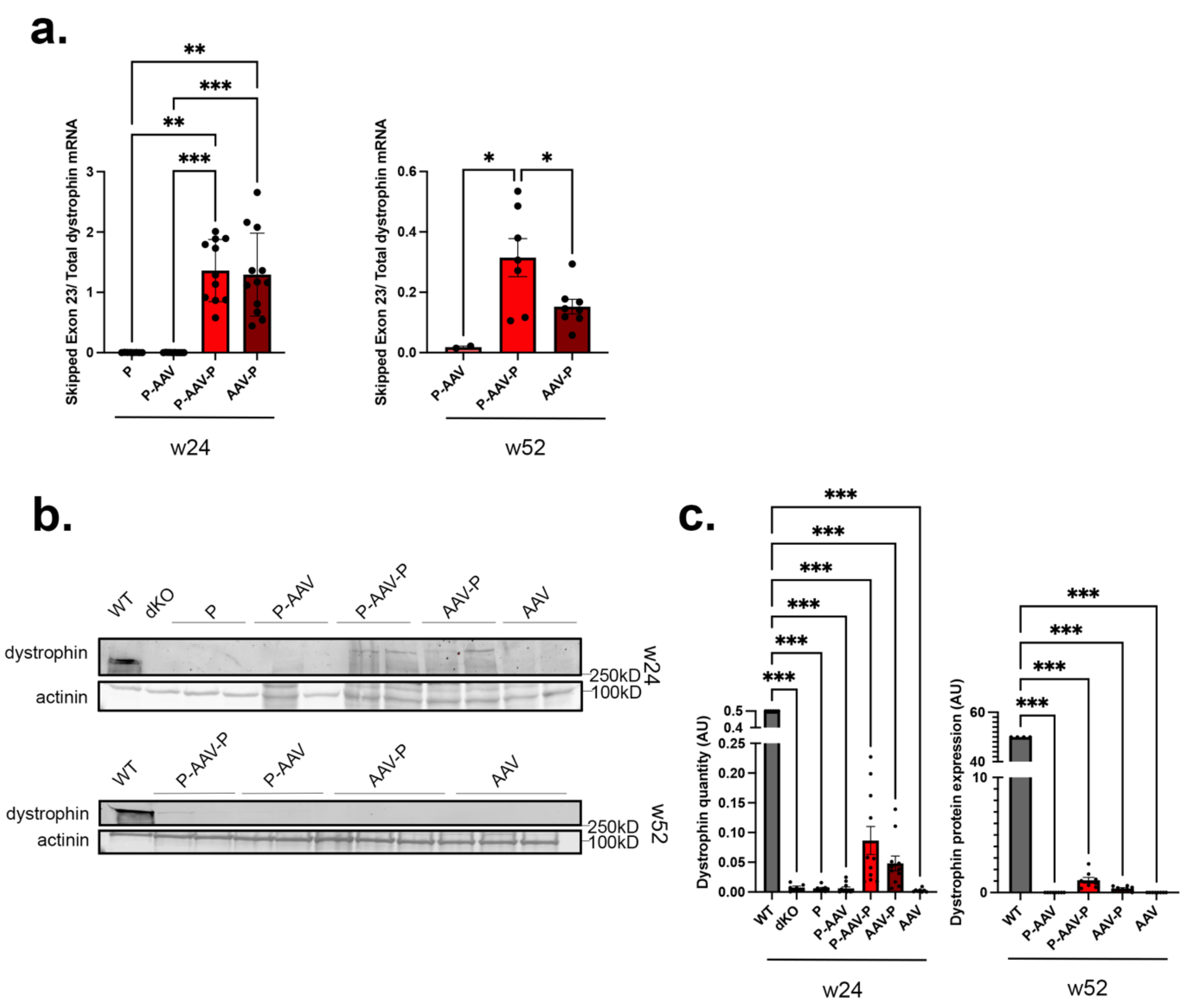

Supplementary Figure 7: (a) Quantification of exon 23 skipped dystrophin gene expression in hearts from 24W and 52W-old dKO treated with PPMO alone (P), PPMO+AAV-micro dystrophin (P-AAV), PPMO+AAV-micro dystrophin+PPMO (P-AAV-P), AAV-micro dystrophin+PPMO (AAV-P) and AAV-micro

dystrophin alone (AAV). Each dot represents one mouse. Data are means ± SEM of at least 8 mice per group. Significance was determined by one-way ANOVA. **(b)** Representative immunoblots showing skipped exon 23 dystrophin expression in hearts from 24W and 52W-old WT mice and dKO mice treated with PPMO alone (P), PPMO+AAV-micro dystrophin (P-AAV), PPMO+AAV-micro dystrophin+PPMO (P-AAV-P), AAV-micro dystrophin+PPMO (AAV-P) and AAV-micro dystrophin alone (AAV). α-actinin was shown as loading control. **(c)** Quantifications of skipped exon 23 dystrophin expression in hearts from 24W and 52W-old dKO treated with PPMO alone (P), PPMO+AAV-micro dystrophin (P-AAV), PPMO+AAV-micro dystrophin+PPMO (P-AAV-P), AAV-micro dystrophin+PPMO (AAV-P) and AAV-micro dystrophin alone (AAV). Graphs are means ± SEM of at least 8 mice per group. Significance was determined by one-way ANOVA with Dunnett's multiple comparison test to WT. (*$p < 0.05$, **$p < 0.01$, ***$p < 0.001$).

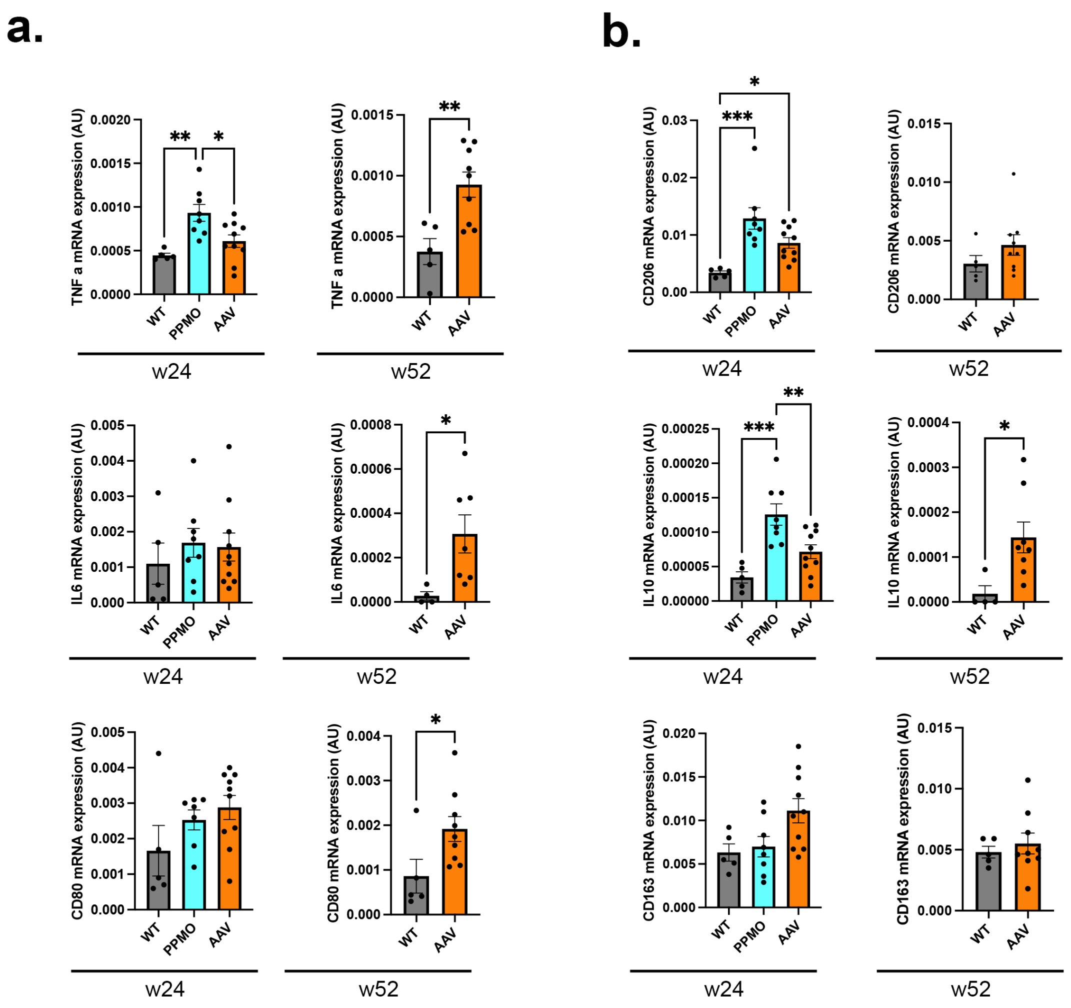

Supplementary Figure 8: (a) Bar graphs showing RT-PCR analysis of TNFα, IL6 and CD80 expression (pro-inflammatory markers). **(b)** Bar graphs showing RT-PCR analysis of CD206, IL10 and CD163 expression (anti-inflammatory markers). Graphs are means ± SEM of at least 4 mice per group.

Significance was determined by one-way ANOVA with multiple comparison test. (*$p < 0.05$, **$p < 0.01$, ***$p < 0.001$).

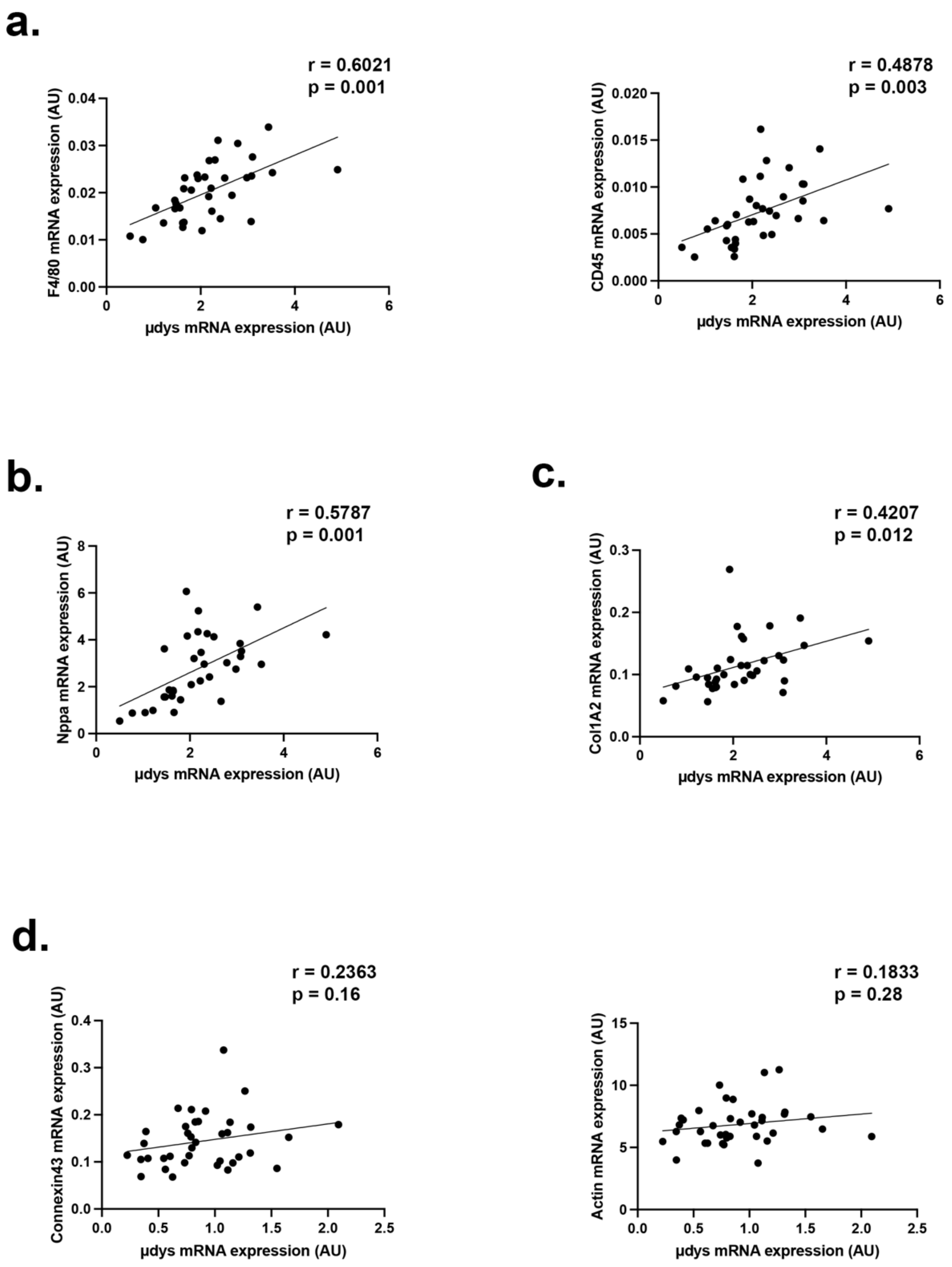

Supplementary Figure 9: (a) Bar graphs showing correlation of micro-dystrophin mRNA expression with *F4/80* mRNA expression (left panel) and *CD45* mRNA expression (right panel). **(b)** Bar graphs showing correlation of micro-dystrophin mRNA expression with *Nppa* mRNA expression. **(c)** Bar graphs

showing correlation of micro-dystrophin mRNA expression with *Col1a2* mRNA expression. **(d)** Bar graphs showing absence of correlation of micro-dystrophin mRNA expression with *Connexin 43 or* α *actin* mRNA expression (negative control). Graphs present data of 35 mice injected with AAV-micro-dystrophin. Significance was determined by Pearson correlation coefficient indicated on the graph.

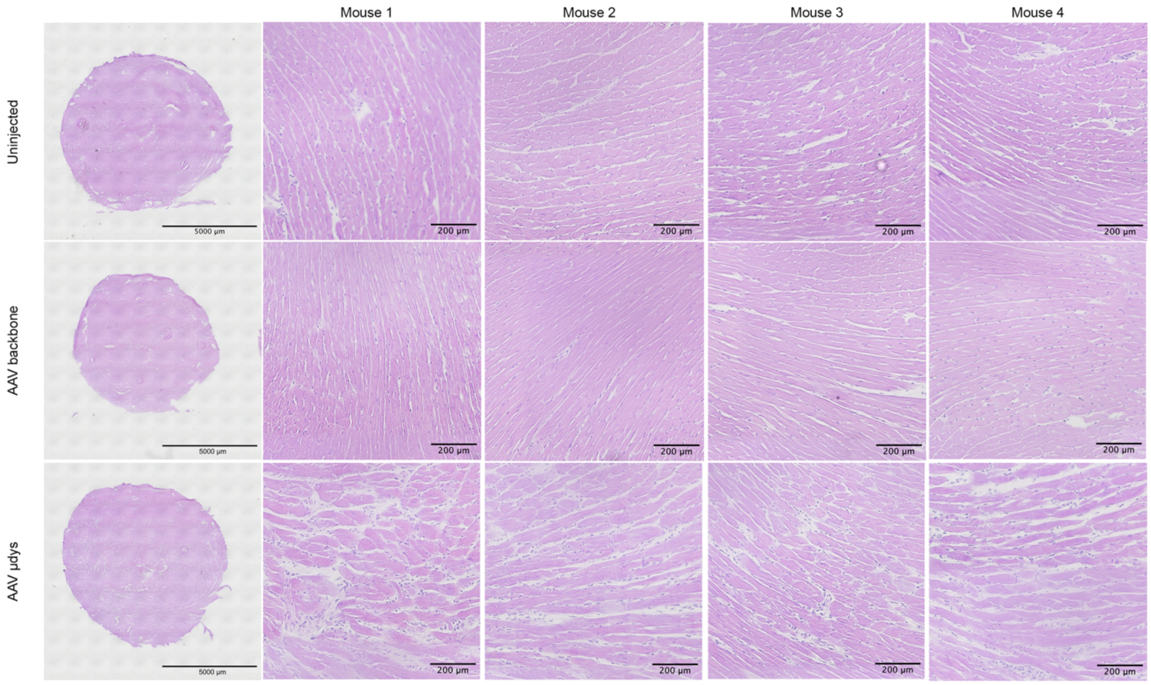

Supplementary Figure 10: Representative micrographs of hematoxylin-eosin-stained sections of heart from 24W-old WT mice non injected or treated with AAV-backbone or AAV-micro dystrophin. 4 mice per group are presented. Scale bars, 200 µm.

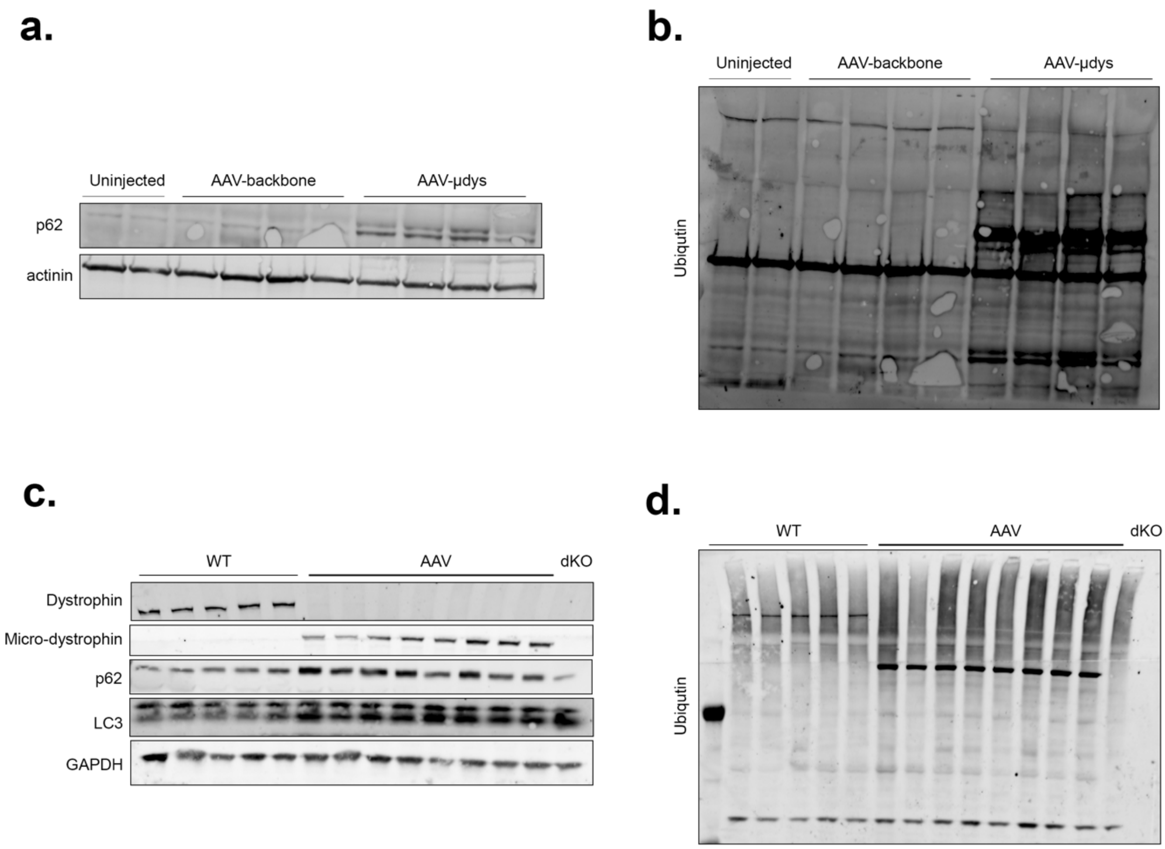

Supplementary Figure 11: (a) Representative immunoblots showing p62 expression in hearts from 24W-old WT mice non injected or treated with AAV-backbone or AAV-micro dystrophin (AAV-µdys). α-actinin was shown as loading control. **(b)** Representative immunoblots showing ubiquitin expression in hearts from 24W-old WT mice non injected or treated with AAV-backbone or AAV-micro dystrophin (AAV-µdys). α-actinin was shown as loading control. **(c)** Representative immunoblots showing dystrophin, micro-dystrophin, p62 and LC3 expression in hearts from 52W-old WT mice and dKO mice treated with AAV alone and 9W old untreated dKO mouse. GAPDH was shown as loading control.

(d) Representative immunoblots showing ubiquitin expression in hearts from 52W-old WT mice and dKO mice treated with AAV alone and 9W old untreated dKO mouse. GAPDH was shown as loading control.

**Metal**	**Antibody**	**Clone**	**Company**	**Dilution**	**Staining**
209Bi	Laminin	4H8-2	AbLab	1:200	ON
173Yb	MHCII	M5/114	AbLab	1:100	ON
172Yb	CD86	GL1	Standard Biotools	1:50	ON
171Yb	Ly6-G	1A8	AbLab	1:200	2h
168Er	CD31	MEC.13.3	AbLab	1:200	2h
166Er	CD90.2	30H12	AbLab	1:100	ON
163Dy	CD54	YN1/1.7.4	Standard Biotools	1:100	ON
159Tb	F4/80	BM8	Standard Biotools	1:50	ON
150 Nd	CD24	M1/69	Standard Biotools	1:100	ON
148Nd	CD11b	M1/70	Standard Biotools	1:50	ON
141Pr	CD146	ME9F1	Standard Biotools	1:200	2h

Table S1 : Antibody panel.

	**WT**	**DKO**	WT-PBS		PPMO-PBS-PBS		PBS-AAV-PBS		PPMO-AAV-PBS		PBS-AAV-PPMO		PPMO-AAV-PPMO	
	**9W**	**9W**	24W	52W	24W	48W	24W	52W	24W	52W	24W	52W	24W	52W
n	**12**	**19**	13	11	8	2	10	10	12	11	12	8	14	8
Time (ms)	**97.0 ± 0.4**	**136.2 ± 7.4*** **	96.3 ± 0.51£££	94 ± 0.8$	120.6 ± 3.1***	102.31 ± 5.8	109.1 ± 2.4***£££	104.7 ± 1.5***	107.9 ± 3.6**£££	104 ± 1.7**	98.1 ± 1.5£££	104.1 ± 2.1**$	102.3 ± 1.4*£££	112.2 ± 3.5***$
Heart rate (Bmp)	**619 ± 3**	**468 ± 14*** **	623 ± 3£££	638 ± 5$	500 ± 13***	588 ± 33	552 ± 12***£	574 ± 9***	561 ± 15***££	578 ± 9***	613 ± 10£££	578 ± 11***$	588 ± 8*£££	538 ± 16***$$
Heart flow (L/min)	**0.058 ± 0.004**	**0.024 ± 0.001*** **	0.053 ± 0.003	0.06 ± 0.01	0.035 ± 0.003	0.045 ± 0.015	0.051 ± 0.01	0.056 ± 0.007	0.051 ± 0.004	0.06 ± 0.003	0.046 ± 0.002	0.06 ± 0.01$	0.051 ± 0.003	0.05 ± 0.01
IVSd (cm)	**0,065 ± 0,001**	**0.053 ± 0.002*** **	0.063 ± 0.001	0.071 ± 0.002$$$	0.061 ± 0.002	0.06 ± 0.01	0.072 ± 0.003***£££	0.091 ± 0.003***$	0.073 ± 0.002*£	0.082 ± 0.003*$	0.068 ± 0.001	0.084 ± 0.003**$$$	0.07 ± 0.002	0.089 ± 0.002***$$$£
LVDd (cm)	**0.356 ± 0.009**	**0.301 ± 0.009*** **	0.340 ± 0.008	0.353 ± 0.013	0.34 ± 0.01	0.41 ± 0.1	0.34 ± 0.01	0.355 ± 0.009	0.34 ± 0.01	0.359 ± 0.009	0.33 ± 0.005	0.363 ± 0.011$$	0.34 ± 0.01	0.344 ± 0.11
LVPWd (cm)	**0.064 ± 0.002**	**0.049 ± 0.002*** **	0.071 ± 0.001	0.073 ± 0.003	0.064 ± 0.006	0.07 ± 0	0.070 ± 0.003	0.084 ± 0.004$	0.074 ± 0.001£	0.086 ± 0.004$$	0.065 ± 0.003	0.085 ± 0.006$	0.076 ± 0.002££	0.085 ± 0.004
IVSs(cm)	**0,109 ± 0,002**	**0.079 ± 0.003*** **	0.112 ± 0.004££	0.124 ± 0.002$	0.095 ± 0.003*	0.09 ± 0.02	0.12 ± 0.01£££	0.151 ± 0.005*$$	0.125 ± 0.004*£££	0.145 ± 0.005	0.12 ± 0.002£££	0.138 ± 0.005$	0.121 ± 0.003£££	0.146 ± 0.003$$$
LVDs (cm)	**0.192 ± 0.006**	**0.183 ± 0.010**	0.185 ± 0.004£££	0.190 ± 0.009	0.2325 ± 0.008***	0.33 ± 0.11	0.182 ± 0.006£££	0.188 ± 0.007	0.1842 ± 0.006£££	0.199 ± 0.006	0.1767 ± 0.003£££	0.204 ± 0.010$	0.1843 ± 0.007£££	0.18 ± 0.008
LVPWs (cm)	**0.103 ± 0.003**	**0.069 ± 0.004*** **	0.1169 ± 0.003£££	0.123 ± 0.005£	0.091 ± 0.007***	0.08 ± 0	0.112 ± 0.006££	0.123 ± 0.007	0.1158 ± 0.003£££	0.129 ± 0.005$	0.1075 ± 0.004£	0.125 ± 0.010£	0.1171 ± 0.003£££	0.129 ± 0.005
Telediastolic volume (ml)	**0.115 ± 0.009**	**0.074 ± 0.007** **	0.1038 ± 0.007	0.115 ± 0.012£	0.1063 ± 0.009	0.205 ± 0.125	0.1050 ± 0.009	0.119 ± 0.009	0.1067 ± 0.008	0.118 ± 0.008£	0.091 ± 0.003	0.123 ± 0.011$$	0.1036 ± 0.008	0.106 ± 0.011
Telesistolic volume (ml)	**0.019 ± 0.002**	**0.020 ± 0.004**	0.018 ± 0.001£££	0.018 ± 0.003	0.0325 ± 0.003***	0.12 ± 0.09	0.014 ± 0.002£££	0.019 ± 0.002	0.0175 ± 0.002£££	0.022 ± 0.002	0.0158 ± 0.001£££	0.023 ± 0.003$	0.018 ± 0.001£££	0.019 ± 0.002
EF(%)	**83,2 ± 0,4**	**76.2 ± 2.3***	83.4 ± 0.3£££	83.3 ± 0.6	67.6 ± 1.9***	48.1 ± 13.93	84.1 ± 0.5£££	83.6 ± 0.8	83.4 ± 0.7£££	82.2 ± 0.8	83.3 ± 0.5£££	81.5 ± 1.1	83.5 ± 0.5£££	84.5 ± 0.9£
SF(%)	**46,0 ± 0,4**	**39.8 ± 1.7***	46.1 ± 0.3£££	46.1 ± 0.6	32.4 ± 1.3***	21.05 ± 7.33	47 ± 0.6£££	46.5 ± 0.9	46.3 ± 0.8£££	45 ± 0.01	46 ± 0.5£££	44.3 ± 1.1	46.3 ± 0.6£££	47.5 ± 1
Ejection volume (ml)	**0.097 ± 0.007**	**0.053 ± 0.003*** **	0.087 ± 0.006	0.096 ± 0.009	0.074 ± 0.007	0.08 ± 0.03	0.089 ± 0.007	0.096 ± 0.007	0.087 ± 0.007	0.099 ± 0.006	0.076 ± 0.003	0.099 ± 0.007$$	0.087 ± 0.006	0.089 ± 0.009
h/r	**0,362 ± 0,010**	**0.345 ± 0.012**	0.39 ± 0.01	0.414 ± 0.01$	0.36 ± 0.01	0.356 ± 0.109	0.42 ± 0.01£	0.49 ± 0.01$$$	0.43 ± 0.01££	0.473 ± 0.013*$$	0.4 ± 0.01	0.464 ± 0.015$$$	0.43 ± 0.01*£££	0.512 ± 0.022***$$
LVDd/body weight (cm/g)	**0,0169 ± 0,0005**	**0.021 ± 0.0002*** **	0.0129 ± 0.0005	0.0115 ± 0.0003$	0.014 ± 0.001	0.019 ± 0.004	0.012 ± 0;001	0.0111 ± 0.0005***	0.0124 ± 0.0004	0.0111 ± 0.0005$	0.008 ± 0.002***£££	0.011 ± 0.001	0.0130 ± 0.0004	0.011 ± 0.0004$$

Table S2: Complete cardiac function parameters: Parameters measured by echocardiography for mice aged 24 and 52 weeks. The data shown are means ± SEM for at least 8 mice (except for the PPMO only treated dKO mice at 48W-old, *n*=2). Significance was determined by one-way ANOVA with Dunnett's multiple comparison test to WT (*) or PPMO group at 24W (£). Student t test was used for comparison between age with the same treatment ($). Data shown for 9W-old WT and dKO are indicative and came from [26].

	WT-PBS		PPMO-PBS-PBS		PBS-AAV-PBS		PPMO-AAV-PBS		PBS-AAV-PPMO		PPMO-AAV-PPMO	
	24W	52W	24W	52W	24W	52W	24W	52W	24W	52W	24W	52W
RR (ms)	83.07 ± 0.86	83.83± 0.6878	100.6 ± 2.654***	ND	91.49 ± 2.289*	89.47 ± 1.37	93.76 ± 3.076**	89.26 ± 1.608	86.84 ± 1.278	87.70 ± 2.184	95.68 ± 5.114	112.2 ± 3.5**
HR	725 ± 8	717 ± 6	603 ± 15***	ND	660 ± 15**	655 ± 19*	652.9 ± 20**	676 ± 12	685 ± 13	688 ± 15	660 ± 13**	644 ± 29*
PR	33.39 ± 0.6537	35 ± 0.7256	33.22 ± 2.006	ND	35.59 ± 1.594	37.61 ± 0.94	33.35 ± 0.859	36.62 ± 1.831	32.86 ± 1.025	38.27 ± 1.406	33.72 ± 1.796	37.51 ± 2.076
QRS	12.62 ± 0.3662	13.11 ± 0.8460	15.29 ± 0.8179	ND	15.62 ± 1.062	14.08 ± 0.5404	15.44 ± 0.7397	14.91 ± 0.8397	14.47 ± 0.4185	12.84 ± 0.5893	16.3 ± 1.2**	14.1 ± 1.004
QT	ND	25.56 ± 0.5879	ND	ND	ND	28.39 ± 1.225	ND	27.8 ± 0.8026	ND	26.66 ± 0.7835	ND	26.78 ± 1.009

Table S3: Complete cardiac function parameters: Parameters measured by electrocardiogragm for mice aged 24 and 52 weeks. The data shown are means ± SEM for at least 8 mice. Significance was determined by one-way ANOVA with Dunnett's multiple comparison test to WT. (* $p < 0.05$, ** $p < 0.01$, *** $p < 0.001$).

Group	n		Inflammation		Necrosis		Mononuclear Cell Infiltration		Fibrosis	
	24W	52W	24W	52W	24W	52W	24W	52W	24W	52W
dKO	16		19%		19%		44%		0	
WT	13	5	0	0	0	0	0	0	0	0
P	8	2	75%	83%	50%	83%	25%	50%	25%	100%
P-AAV	12	11	0	82%	0	82%	83%	82%	0	91%
P-AAV-P	14	8	14%	63%	14%	75%	93%	50%	0	75%
AAV-P	11	8	0	75%	0	75%	73%	88%	9%	63%
AAV	10	11	0	82%	0	82%	80%	91%	0	100%

Table S4: Quantification of histopathological features of inflammation in heart sections from 24 and 52W-old WT and dKO mice treated with PPMO alone (P), PPMO+AAV-micro dystrophin (P-AAV), PPMO+AAV-micro dystrophin+PPMO (P-AAV-P), AAV-micro dystrophin+PPMO (AAV-P) and AAV-micro dystrophin alone (AAV) and from 9W-old untreated dKO mice.

Table S5: Microarray data comparing 52W-old untreated WT and AAV treated dKO hearts.

Table S6: Enrichment analysis comparing 52W-old untreated WT and AAV treated dKO hearts using hallmark gene sets collection.

	WT non injectd		WT AAV backbone		WT AAV µdys	
	12W	24W	12W	24W	12W	24W
n	10	7	4	4	4	4
Time (ms)	97.81 ± 0.09	97,59 ± 1.13	99,17 ± 1.7	97,545 ± 1.4	105,75 ± 3.73*	98,66 ± 3
Heart rate (Bmp)	613 ± 0.6	615 ± 7.1	606 ± 10.5	616 ± 9.3	570 ± 20.5	610 ± 18.2
Heart flow (L/min)	0.0525 ± 0.003	0,0514 ± 0.004	0,0575 ± 0.008	0,0475 ± 0.003	0,04 ± 0.004*	0,0325 ± 0.003**
IVSd (cm)	0.0625 ± 0.002	0,065 ± 0.002	0,07 ± 0.004	0,07 ± 0.007	0,0825 ± 0.003**	0,1025 ± 0.003****
LVDd (cm)	0.3425 ± 0.005	0,34 ± 0.01	0,35 ± 0.017	0,3275 ± 0.009	0,32 ± 0.011	0,2825 ± 0.006**
LVPWd (cm)	0.07 ± 0.002	0,08 ± 0.004	0,075 ± 0.003	0,075 ± 0.003	0,08 ± 0.006	0,09 ± 0.007
IVSs(cm)	0.1125 ± 0.002	0,121 ± 0.003	0,1325 ± 0.005	0,1225 ± 0.003	0,1375 ± 0.003	0,155 ± 0.003****
LVDs (cm)	0.185 ± 0.002	0,183 ± 0.006	0,18 ± 0.013	0,175 ± 0.009	0,155 ± 0.009*	0,14 ± 0.007**
LVPWs (cm)	0.1175 ± 0.005	0,124 ± 0.006	0,12 ± 0.009	0,12 ± 0.007	0,1225 ± 0.005	0,1275 ± 0.009
Telediastolic volume (ml)	0.105 ± 0.004	0,103 ± 0.008	0,11 ± 0.017	0,09 ± 0.007	0,0875 ± 0.006	0,06 ± 0.004
Telesistolic volume (ml)	0.02 ± 0.000	0,016 ± 0.003	0,0175 ± 0.005	0,0175 ± 0.003	0,01 ± 0.000	0,01 ± 0.000**
EF(%)	83.2± 0.3	83,6 ± 1	85,6 ± 1.4	84,4 ± 0.7	87,8 ± 1*	87,2 ± 0.8*
SF(%)	46 ± 0.3	46,4 ± 1.1	48,85 ± 1.8	47,3 ± 0.8	51,5 ± 1.3*	50,6 ± 1*
Ejection volume (ml)	0.0875 ± 0.004	0,086 ± 0.008	0,095 ± 0.012	0,0775 ± 0.006	0,0725 ± 0.005	0,0525 ± 0.003*
h/r	0.39075 ± 0.004	0,42 ± 0.014	0,4135 ± 0.021	0,43925 ± 0.02	0,498 ± 0.023***	0,6755 ± 0.024****

	WT non injectd		WT AAV backbone		WT AAV µdys	
	12W	24W	12W	24W	12W	24W
RR (ms)	82.5 ± 1.44	80.6 ± 0.89	80.2 ± 1.21	79.3 ± 0.81	84.3 ± 1.42	81.3 ± 1.88
HR	728.8 ± 12.35	746.1 ± 7.84	748.9 ± 11.37	756.9 ± 7.68	712.9 ± 12.22	742.2 ± 19.92
PR	33.8 ± 0.49	32.7 ± 0.88	32.2 ± 0.83	32.0 ± 0.64	32.6 ± 0.35	33.1 ± 0.36
QRS	11.4 ± 0.24	11.7 ± 0.26	11.7 ± 0.24	11.9 ± 0.27	14.0 ± 0.68**	14.4 ± 0.22****
QT	22.4 ± 0.43	22.1 ± 0.34	22.5 ± 0.38	23.1 ± 0.8	25.6 ± 1.48*	24.6 ± 1.1*

Table S7: **Complete cardiac function parameters:** Parameters measured by echocardiography (upper table) for 24W old WT mice non injected or treated with AAV-backbone or AAV-micro-dystrophin. The data shown are means ± SEM for at least 4 mice. Significance was determined by one-way ANOVA with Dunnett's multiple comparison test to WT. Parameters measured by electrocardiogragm (lower panel) for 24W old WT mice non injected or treated with AAV-backbone or AAV-micro-dystrophin. The data shown are means ± SEM for at least 4 mice. Significance was determined by one-way ANOVA with Dunnett's multiple comparison test to WT. (*p < 0.05, **p < 0.01, ***p < 0.001, ****p < 0.0001).

26. Forand, A. *et al.* Combined Treatment with Peptide-Conjugated Phosphorodiamidate Morpholino Oligomer-PPMO and AAV-U7 Rescues the Severe DMD Phenotype in Mice. *Mol Ther Methods Clin Dev* 17, 695–708 (2020).
